# Characterization of Porcine Hepatic and Intestinal Drug Metabolizing CYP450: Comparison with Human Orthologues from A Quantitative, Activity and Selectivity Perspective

**DOI:** 10.1038/s41598-019-45212-0

**Published:** 2019-06-25

**Authors:** Wim Schelstraete, Laura De Clerck, Elisabeth Govaert, Joske Millecam, Mathias Devreese, Dieter Deforce, Jan Van Bocxlaer, Siska Croubels

**Affiliations:** 1 Department of Pharmacology, Toxicology and Biochemistry, Laboratory of Pharmacology and Toxicology, Faculty of Veterinary Medicine, Ghent University, Salisburylaan 133, 9820 Merelbeke, Belgium; 2 Department of Pharmaceutics, Laboratory of Pharmaceutical Biotechnology, Faculty of Pharmaceutical Sciences, Ghent University, Ottergemsesteenweg 460, 9000 Gent, Belgium; 3 Department of Bioanalysis, Laboratory of Medical Biochemistry and Clinical Analysis, Faculty of Pharmaceutical Sciences, Ghent University, Ottergemsesteenweg 460, 9000 Gent, Belgium

**Keywords:** Oxidoreductases, Proteomic analysis, Preclinical research

## Abstract

Over the past two decades, the pig has gained attention as a potential model for human drug metabolism. Cytochrome P450 enzymes (CYP450), a superfamily of biotransformation enzymes, are pivotal in drug metabolism. Porcine CYP450 has been demonstrated to convert typical substrates of human CYP450. Nevertheless, knowledge and insight into porcine CYP450 quantity and substrate selectivity is scant, especially regarding intestinal CYP450. The current study aimed to map the quantities of hepatic and intestinal CYP450 in the conventional pig by using a proteomic approach. Moreover, the selectivity of the six most common used probe substrates (phenacetin, coumarin, midazolam, tolbutamide, dextromethorphan, and chlorzoxazone) for drug metabolizing enzyme subfamilies (CYP1A, CYP2A, CYP3A, CYP2C, CYP2D and CYP2E respectively), was investigated. Hepatic relative quantities were 4% (CYP1A), 31% (CYP2A), 14% (CYP3A), 10% (CYP2C), 28% (CYP2D) and 13% (CYP2E), whereas for the intestine only duodenal CYP450 could be determined with 88% for CYP3A and 12% for CYP2C. Furthermore, the results indicate that coumarin (CYP2A), midazolam (CYP3A), tolbutamide (CYP2C), and dextromethorphan (CYP2D) are as selective for porcine as for human CYP450. However, phenacetin (CYP1A2) and chlorzoxazone (CYP2E1) are less selective for the specific enzyme, despite similarities in selectivity towards the different enzymes involved compared to humans.

## Introduction

Up to 55% of the available drugs in human medicine are metabolized by cytochrome P450 (CYP450) enzymes. These enzymes are major contributors to phase I drug metabolism and catalyze oxidative, reductive, and hydrolytic reactions of endogenous and xenobiotic compounds^1,2^. The resulting compounds are often more water soluble and available for further phase II conjugation^3,4^. Regarding xenobiotic metabolism, members belonging to the CYP1, CYP2 and CYP3 family are crucial, and are responsible for 70 to 80% of CYP450 mediated drug metabolism^2,3,5^. Hence, CYP450 biotransformation is investigated early on in the development of new drugs.

The establishment of a preclinical animal model that correlates well with humans, is a valuable tool in drug development and safety testing^6^. However, frequently used animals such as the mouse, rat and rabbit, are often not ideal for modelling human drug metabolism, possibly due to differences in enzyme expression and/or selectivity^7–10^. In the past two decades the pig has gained attention as a more appropriate animal model for humans due to similar anatomy and physiology^11–14^. In pigs, orthologues of human CYP1A2, CYP2A6, CYP3A4/5, CYP2C9/19, CYP2D6, and CYP2E1 have been isolated and identified with homologies in amino acid sequence, ranging from 62 to 87.5%^15^. Sequence identity does not guarantee similar activity/selectivity though. Single nucleotide polymorphisms have been shown to significantly alter CYP450 expression, stability, and function^16,17^. For example, recent insights in the two CYP2C9 polymorphisms show differences in side chain interaction, ultimately leading to different binding of the substrate losartan^18^.

In previous research both pigs and minipigs have been used as a model for humans. Unfortunately, very few studies consistently compared minipig CYP450 expression (and activity) vs. conventional pig CYP450, hence the use of the word ‘(mini)pig’ in the text below^19–22^. The detailed comparison and distinction between the two types of pigs is further complicated by a lack of quantitative data of pig and minipig CYP450 expression at the protein level. The few reports available indicate that differences in activity between conventional and minipigs primarily originate from differences in immunochemically quantified CYP450 enzymes^19,22^, stressing the importance of comparing activities from a quantitative perspective. Furthermore, CYP450 amino acid sequence in conventional and minipigs seems to be almost identical. Specifically there is only one amino acid difference in the CYP2E1 enzyme and a predicted difference of 10 amino acids between minipig CYP2D21 and conventional porcine CYP2D25^15,23^, however the latter has not been verified at the protein level.

When comparing porcine and human CYP450, three main areas of interest can be distinguished: mRNA expression, enzyme quantity, and CYP450 activities.

To date, mRNA expression of (mini)pig CYP450 has been determined in the liver, intestine, lung, kidney, heart, and even brain^24–35^. Although mRNA expression data can be indicative of the amount of translated protein, mRNA levels are not predictive for protein quantity and activity. This can be due to mRNA instability, protein degradation, and translational or posttranslational modifications. Consequently, direct comparison between mRNA amounts are of limited use^36^.

Cytochrome P450 proteins have usually been quantified by Western blotting, resulting in relative expressions of the investigated enzymes^19,37,38^. Although Western blotting has proven to be a valuable tool, it has the limitation that a selection of enzymes of interest has to be made prior to the experiments. Contrarily, a mass spectrometry based method does not impose such prior restrictions and allows for absolute quantification more easily. Such an approach was used by Achour *et al*. (2011) to quantify hepatic CYP450 in conventional (Suffolk White) adult pigs by a state-of-the-art high resolution mass spectrometry (HRMS) method^39^. Unfortunately, the study was performed on just two pigs and did not include quantification of intestinal CYP450 enzymes. Moreover, intestinal quantitative data are restricted to CYP1A1 and CYP3A enzymes^33,40,41^ of which none included the entire small intestine, i.e. duodenum, jejunum, and ileum. Nonetheless, this information is pivotal for *in vitro-in vivo* correlations and to successfully determine the contribution of each enzyme to specific biotransformation reactions^42^.

In the past, typical probe substrates for human CYP450 have been used to measure CYP450 activity in the (mini)pig. Phenacetin (CYP1A), coumarin (CYP2A), midazolam (CYP3A), tolbutamide (CYP2C), dextromethorphan (CYP2D), and chlorzoxazone (CYP2E) are commonly used substrates in human drug research^43^. These substrates are also metabolized by CYP450 in the (mini)pig^19,20,37,44–50^. Although similarities and differences in biotransformation rates between humans and pigs have been discussed^15,51^, less attention has been devoted to the apparent selectivity of the substrates used. It has been shown that chlorzoxazone is metabolized by porcine recombinant CYP2A, CYP1A, and CYP2C enzymes, although it is a typical CYP2E1 substrate^47,52^. Furthermore, it has been proposed that dextromethorphan-O-demethylation may be catalyzed by CYP2B in the pig rather than CYP2D^20^, even though no CYP2B22 was found in conventional pigs^39^.

Therefore, the goals of the current study were (1) to consistently map and characterize the hepatic and intestinal CYP450 enzymes in 16 conventional, 12 weeks old pigs, to allow a more rational comparison between pigs and minipigs in future research, (2) to assess the apparent selectivity of the most commonly used probe substrates towards six important CYP450 enzymes for drug metabolism, which are phenacetin (CYP1A), coumarin (CYP2A), midazolam (CYP3A), tolbutamide (CYP2C), dextromethorphan (CYP2D), and chlorzoxazone (CYP2E), and 3) to compare porcine and human CYP450 with respect to substrate selectivity and activity.

## Materials and Methods

### Chemicals and reagents

Phenacetin (PH), acetaminophen or paracetamol (PAR), tolbutamide (TB), 7-hydroxy-coumarin (OH-CM), dextrorphan-D3 (DXT-D3), coumarin (CM), chlorzoxazone (CZ), diethyldithiocarbamate (DDC), ketoconazole (KET), α-naphthoflavone (α-NFV), 8-methoxypsoralen (8-MPS), protease inhibitor (cOmpleteTM Protease Inhibitor Cocktail, Roche), trifluoroacetic acid (TFA), dimethylsulfoxide (DMSO), triethylammonium bicarbonate (TEABC), dithiotreitol (DTT), methyl methane thiosulfonate (MMTS), calcium chloride, and phosphate buffered saline (PBS) were purchased from Sigma Aldrich (St. Louis, MO, USA). Midazolam (MDZ), 1-hydroxy-midazolam (OH-MDZ), 1-hydroxy-midazolam-D4 (OH-MDZ-D4), 4-hydroxy-tolbutamide (OH-TB), 6-hydroxy-chlorzoxazone (OH-CZ) were obtained from LGC standards (Molsheim, France). Dextromethorphan (DXM), dextrorphan tartrate (DXT), 7-hydroxycoumarin-D5 (OH-CM-D5), acetaminophen-D4 (PAR-D4), 4-hydroxytolbutamide-D9 (OH-TB-D9), quinidine (QND) and sulphaphenazole (SFZ) were purchased from Toronto Research Chemicals (North York, ON, Canada). NADPH was obtained from OYC Europe (Rotterdam, The Netherlands). Six-hydroxy-chlorzoxazone-^13^C_6_ (OH-CZ-^13^C_6_) was obtained from Alsachim (Illkirch Graffenstaden, France). Potassium chloride, potassium dihydrogenphosphate and dipotassium hydrogenphosphate, citric acid, glycerol, disodium hydrogenphosphate, and EDTA were obtained from VWR (Leuven, Belgium). Acetonitrile (ACN), methanol (MeOH), ethylacetate were of HPLC grade and purchased from Fisher Chemicals.

Stock solutions of each substrate and inhibitor were prepared in MeOH (MDZ, 3.26 mg/mL; CM, 0.13 mg/mL; DXM, 6.67 mg/mL; PH, 16.14 mg/mL; KET, 0.32 mg/mL; 8-MPS, 0.43 mg/mL; QND, 0.07 mg/mL; α-NFV, 0.06 mg/mL) or ACN (TB, 48.66 mg/mL; CZ, 15.26 mg/mL; SFZ, 0.32 mg/mL; DDC, 9.01 mg/mL)) and stored at −20 °C. Fresh working solutions were prepared by adding an appropriate amount of stock solution to HPLC-quality water. The stop reagent consisted of 55% ACN, 42% HPLC water, and 3% formic acid with internal standards (final concentrations: 40 ng/mL, 100 ng/mL, 100 ng/mL, 200 ng/mL, 200 ng/mL and 40 ng/mL for OH-MDZ-D4, OH-CZ-^13^C_6_, OH-TB-D9, OH-CM-D5, PAR-D4 and DXT-D3 respectively).

### Preparation of microsomes

Microsomes were prepared from hepatic and intestinal tissues collected from sixteen different conventional pigs (hybrid sow × Piétrain boars, 12 weeks of age, 8 boars and 8 sows). Pigs of this age were selected as most human CYP450 enzymes reach adult activity at the age of 6 years, which corresponds to an age of 4–12 weeks in pigs^53,54^. All procedures were in accordance with the ethical standards of the ethical committee of the Faculties of Veterinary Medicine and Bioscience Engineering of Ghent University (approval EC2015_213).

Fresh samples were taken from the left liver lobe, mid-duodenum, mid-jejunum and mid-ileum, rinsed in PBS and immediately snap frozen in liquid nitrogen and stored at −80 °C. Hepatic microsomes were prepared according to Wilson *et al*.^55^ and the intestinal microsomes were prepared using the mincing method described by Osselaere *et al*.^56^.

Briefly, liver tissues, stored for a maximum of 8 weeks, were thawed on ice in approximately 4 mL of a 0.25 M phosphate buffer (pH 7.25) containing 1.15% aqueous KCl (buffer A). Next, 4 grams of tissue was transferred to a petri dish and minced into small pieces, subsequently transferred to a Potter Elvehjem homogenizer and homogenized in 8 mL of buffer A. Glass tubes were rinsed twice with 4 mL of the same buffer solution and added to the homogenate. Samples were then centrifuged at 10,000 × *g* for 25 min at 4 °C. After centrifugation, the supernatant was transferred to ultracentrifugation tubes and centrifuged at 100,000 × *g* for 80 min at 4 °C. Samples were washed 4 times with 3 mL of buffer A solution and again centrifuged at 100,000 × *g* for 80 min at 4 °C. The microsomal pellets were suspended in 1.5 mL/g liver tissue of buffer A containing 30% glycerol, snap frozen and stored at −80 °C. All procedures were performed on ice.

Intestinal tissues were prepared according to an analogous procedure. Four gram of each segment was thawed on ice in 4 mL of thaw solution containing 1.5 mM KCl, 96 mM NaCl, 27 mM sodium citrate, 8 mM KH_2_PO_4_, 5.6 mM Na_2_HPO_4_ and one tablet of protease inhibitor per 50 mL of solution. After mincing, the tissues were transferred to a Potter Elvehjem homogenizer. Tissues were homogenized in 8 mL of homogenization buffer (50 mM phosphate buffer with 1 mM EDTA and 1 tablet of protease inhibitor per 50 mL solution). Glass tubes were rinsed twice with 4 mL of homogenization buffer. Samples were centrifuged at 18,000 × *g* for 20 min after which the supernatant was transferred and centrifuged at 100,000 × *g* for 67 min at 4 °C. Microsomal pellets were washed with a 125 mM phosphate buffer (pH 7.5 containing 1.25 mM EDTA and 20% glycerol, resuspension buffer) and centrifuged for a second time at the conditions specified. Afterwards, microsomal pellets were suspended in 0.5 mL/gram tissue of resuspension buffer, snap frozen and stored at −80 °C. All procedures were performed on ice.

Hepatic and intestinal microsomal protein concentrations were determined by the Bradford assay^57^, according to the manufacturer’s instructions.

### CYP450 quantitative measurement using proteomics

#### HD-DDA MS experimental set-up

Microsomal proteins (20 µg) were reduced in 0.5 M TEABC and 1 mM DTT for 1 hour at 60 °C, followed by alkylation using 10 mM MMTS for 10 min at room temperature. Proteins were digested into peptides using trypsin (33:1 protein/enzyme ratio; Promega, Wisconsin, USA) overnight at 37 °C with CaCl_2_ and ACN to a final concentration of 1 mM and 5%, respectively. After evaporation in a speedvac, the samples were re-suspended in 0.1% formic acid. Four hundred ng sample was spiked with 50 fmol β-galactosidase (Sciex, Washington DC, USA) and 50 fmol Hi3 *Escherichia coli* (Waters, Massachusetts, USA) standards before injection. For the intestinal microsomes, samples of all sixteen pigs were pooled and measurements were performed in triplicate. Hepatic CYP450 quantity was determined for each pig.

The peptides were separated using a nanoscale UPLC system (nanoAcquityUPLC, Waters, Milford, USA) coupled to a HRMS Q-TOF Synapt G2-Si mass spectrometer (Waters, Massachusetts, USA). Peptides were first trapped in 0.1% formic acid on a 180 µm × 20 mm C18 Trap column. Separation was performed on a HSS C18 1.8 µm, 75 µm × 250 mm analytical column at a flow rate of 300 nL/min and a temperature of 45 °C. Mobile phase A and B were composed of 0.1% formic acid with 4% DMSO in UPLC-water and 80% ACN containing 0.1% formic acid, respectively. Peptides were separated with a linear gradient for 60 min at 1–40% solvent B and for 1 min at 40–85% solvent B. The HRMS instrument was operated in positive mode for High Definition-DDA, using a nano-ESI source, acquiring full scan MS and MS/MS spectra (*m/z* at 50–5,000) in resolution mode. Survey MS scans were acquired using a fixed scan time of 200 ms. Tandem mass spectra of up to eight precursor ions with charge state 2+ to 5+ were generated using CID in the trapping region with intensity threshold set at 3,000 cps, using a collision energy ramp from 6/9 V (low mass, start/end) up to 147/183 V (high mass, start/end). MS/MS scan time was set to 100 ms with an accumulated ion count ‘TIC stop parameter’ of 100,000 cps allowing a maximum accumulation time of 250 ms. Dynamic exclusion of fragmented precursor ions was set to 12 s. Ion mobility spectrometry wave velocity was ramped from 2,500 to 400 m/s. Wideband enhancement was used to obtain a near-100% duty cycle on singly-charged fragment ions. LockSpray of glufibrinopeptide-B (*m/z* 785.8427) was acquired at a scan frequency of 60 s.

#### Data-analysis

Data analysis of the raw files obtained from the Synapt G2-Si was performed in Progenesis® QI (Nonlinear Dynamics) version 2.3. Peptides with charge +1 were discarded. For relative quantification, data was normalized to all proteins. For absolute quantification, data was normalized to Hi3 *E*. *coli* peptides. Peptide identification was performed with Mascot 2.5, the following search criteria were set: trypsin as digestion enzyme, up to two missed cleavages allowed, fixed modification of methylthiocysteine and variable modifications of methionine oxidation and deamidation at asparagine and glutamine. Peptide mass tolerance was set to 15 ppm and fragment mass tolerance to 0.2 Da. Protein identifications were obtained by searching a compiled database of reviewed *Sus Scrofa* entries (Swissprot), supplemented with unreviewed CYP proteins and fragments of interest, the cRAP database (laboratory proteins and dust/contact proteins, http://www.thegpm.org/crap/), and sequences of spiked standard proteins. For relative quantification, the top three peptides were used and only proteins with at least one unique peptide were further considered. For absolute quantification, proteins were quantified using the top three peptides against Hi3 *E*. *coli* peptides, and only proteins with at least one unique peptide were further considered. Although it is hard to assume that the ionization efficiency is the same for each peptide and in each sample, this method has been proven reproducible (10% CV) and accurate (15% relative error) for the purpose of comparison^58^. Protein data was exported from Progenesis® for further statistical analysis. For the calculations of relative quantity of drug metabolizing CYP450, the mean quantity of each enzyme was calculated as the percentage of the mean total detected drug metabolizing enzyme.

### CYP450 enzyme activity measurements and inhibition experiments

#### Activity measurements

Hepatic microsomes were incubated at 37 °C with TB, CZ, MDZ, CM, DXM, and PH at six different concentrations (Table 1). The incubation times were derived from linearity experiments. Incubation were performed with different protein concentrations (0.1, 0.25, 0.5, and 0.75 mg/mL) and incubation times (5, 10, 20, 30, and 45 min) to determine the linear biotransformation area (data not shown). The substrate concentrations used were 100 µM, 20 µM, 1 µM, 20 µM, 5 µM, and 5 µM for TB, CZ, CM, PH, MDZ, and DXM respectively. Protein concentrations and time were chosen at the lowest possible value to allow reliable quantification, within the linear range of the biotransformation reaction.

**Table 1:** Incubation conditions for the establishment of concentration-activity profiles in porcine hepatic microsomes.

Probe	Enzyme subfamily	Final protein concentration (mg/mL)	Incubation time^*^ (min)	Concentration range (µM) (6 levels)
Tolbutamide	CYP2C	0.25	10	5.00–10.0–50.0–100–200–400
Chlorzoxazone	CYP2E	0.1	5	2.50–5.00–25.0–50.0–100–200
Coumarin	CYP2A	0.1	5	0.25–0.50–2.00–5.00–20.0–100
Phenacetin	CYP1A	0.1	5	1.00–5.00–20.0–50.0–100–200
Midazolam	CYP3A	0.1	5	0.50–2.00–5.00–10.0–20.0–50.0
Dextromethorphan	CYP2D	0.1	5	0.10–0.50–1.00–5.00–20.0–100

After the specified incubation time, an aliquot of 200 µL was transferred to an Eppendorf tube containing 125 µL of ice cold stop reagent. Samples of the same animal, incubated with different probes, were pooled for each concentration and 125 µL of TFA was added. Subsequently, samples were centrifuged for 10 min at 16,200 × *g* at 4 °C. Following centrifugation, the supernatant was transferred to 15 mL conical tubes containing 1 mL of a 100 mM phosphate buffer. Next, 7 mL of ethylacetate was added and samples were extracted for 15 min on an overhead shaker (IKA® TRAYSTER, Staufen, Germany). The two phases were separated by centrifuging at 14,000 × *g* for 5 min. The organic phase was transferred and evaporated at 40 ± 5 °C under a gentle nitrogen stream. Samples were reconstituted in 200 µL of a 50/50 methanol/water solution and analyzed by LC-MS/MS as previously described^59^.

Intestinal microsomes were incubated as described above for hepatic microsomes. The final microsomal protein concentration was 0.75 mg/mL and incubation time was 10 min for all of the probe reactions. Each activity measurement (hepatic and intestinal) was performed in triplicate and data are presented as mean of these replicates.

#### Michaelis-Menten, Hill kinetics, and Eadie-Hofstee plots

The activity data were fitted against the standard Michaelis-Menten equation (Eq. (1a), first term). For each profile, Eadie-Hofstee plots were derived to investigate involvement of more than one enzyme or deviation from normal Michaelis-Menten behaviour. If the Eadie-Hofstee plot was hyperbolic, the data was fitted against the Hill equation^60^. K_m_, V_max_ and/or Hill coefficients were derived for each substrate and pig, in order to obtain the inter-individual variation.

#### Incubation with inhibitors and two substrates

To assess the selectivity of the probes, incubation with human specific CYP450 inhibitors was performed. The inhibitors used were α-NFV, 8-MPS, KET, SFZ, QND, and DDC for CYP1A/2 A, CYP2A, CYP3A, CYP2C, CYP2D, and CYP2E1, respectively. Each inhibitor was evaluated with its corresponding substrate at concentrations around the K_m_ value (20 µM, 1 µM, 20 µM, 200 µM, 10 µM and 50 µM for PH, CM, MDZ, TB, DXM and CZ, respectively). Considering the goal of this study, the concentrations of inhibitors were chosen to be 0.1 µM, 1 µM, 0.3 µM, 0.5 µM, 0.1 µM and 20 µM for α-NFV, 8-MPS, KET, SFZ, QND, and DDC respectively (Supplementary Table 4). These values correspond to the K_i_ values towards human CYP450 orthologues, assuming relative similar inhibition for human and porcine CYP450 orthologues. All incubations were performed in triplicate.

Second, two substrates were incubated simultaneously to investigate their influence on each other’s biotransformation rate. For the dual substrate incubation experiment, each possible combination of two substrates was investigated and incubated at concentrations around or below the K_m_ value. All incubations were performed in triplicate. From these experiments, rate ratios were calculated as Rate_(substrate + inhibitor)_/Rate_(control)_ and Rate_(dual substrate)_/Rate_(control)_. The incubations with inhibitors and two substrates were only performed on porcine hepatic microsomes, due to the limited availability and low activity of porcine intestinal microsomes.

### Selectivity assessment of probes towards porcine CYP450

First, Eadie-Hofstee plots were examined for deviations from linearity. Biphasic plots are indicative of multiple enzyme involvement or of the presence of multiple binding sites within the enzyme. Furthermore, correlations between substrate-enzyme and substrate-substrate were analyzed to investigate changes in explained variability. In essence, at a fixed concentration, enzyme kinetics can be approximated by a linear function of enzyme concentration(s) (Eqs (1a–d)). Changes in correlation coefficients are therefore a reflection of a changing contribution of the enzyme(s) involved in the biotransformation.

Second, to identify CYP450 enzymes involved in the biotransformation, stepwise multiple linear regression was performed. For each typical reaction, the activity data at low concentrations (2^nd^ lowest concentration, Table 1) and high concentration (highest concentration, Table 1) were set as the dependent variable to make a qualitative distinction between low and high affinity CYP450 enzymes. Independent variables were CYP450 protein concentrations as measured by the HRMS method. The criterion for inclusion of the independent variable in the model was a probability of the F-statistic <0.1, while the criterion for exclusion was a probability of the F-statistic >0.2. This approach can be rationalized by assuming that theoretically the Michaelis-Menten constant, K_m_, is a constant over all individuals. This assumption is reasonable as no polymorphisms are known to date that affect the intrinsic clearance of substrate drugs^15,44^. Nevertheless, it should be noted that one polymorphism (1423 G- > A) in porcine CYP2E1 can lead to a decrease in protein expression^61^. However, it remains unclear if this polymorphism results in the alteration of the enzyme’s metabolic properties due to conflicting results^62,63^. Given this and a fixed concentration for each reaction, the Michaelis-Menten equation (Eq. (1a)) can be reduced to the Eq. (1d) depicted below.

1a $$\frac{dv}{dt}=\frac{[S]\ast {V}_{max{\rm{\_}}1}}{{K}_{m{\rm{\_}}1}+[S]}+\frac{[S]\ast {V}_{max{\rm{\_}}2}}{{K}_{m{\rm{\_}}2}+[S]}+\ldots +\frac{[S]\ast {V}_{max{\rm{\_}}n}}{{K}_{m{\rm{\_}}n}+[S]}+Constant$$

1b $${V}_{max\_n}={k}_{cat\_n}\ast {[E]}_{n}$$

1c $$\frac{[S]\ast {k}_{cat\_n}}{{K}_{m\_n}+[S]}={\theta }_{n}$$

1d $$\frac{dv}{dt}={\theta }_{1}{[E]}_{1}+{\theta }_{2}\,{[E]}_{2}+\ldots +{\theta }_{n}{[E]}_{n}+Constant$$

Where *dv/dt*, *V*_*max*_, *K*_*m*_, *k*_*cat*_, $\,\theta$, *[E]*, and *[S]* are the reaction rate, maximal reaction rate, Michaelis-Menten constant, catalytic constant, linear coefficients, enzyme concentration, and substrate concentration respectively. Using the same approach, activity data of the substrates were used as independent variables in a second stepwise multiple linear regression analysis. This approach can be rationalized by assuming that two probes, which are metabolized by the same enzyme, will show a strong correlation and thus explain each other’s variability. Only variables associated with positive coefficients, were retained for further investigation.

Since enzymes can be highly correlated due to co-regulation, collinearity was an anticipated problem. To control the collinearity and thus the stability of the regression, independent variables were standardized. Tolerance values were examined and variables with values below 0.3 were considered for further investigation, in addition to the variables included in the model. Variables selected this way will be named collinear enzymes or collinear metabolites.

Third, inhibition of the substrates on each other’s biotransformation rate was investigated to detect shared enzymes. Since most of the substrates and/or their metabolites will inhibit a reaction to a certain extent and taking into account the accuracy of the used quantification method, which has bounds between −20% and +10%, an inhibition of at least 20% was deemed relevant.

Fourth, typical inhibitors for each of the reactions were included at previously reported K_i_ values^43,64^. The same boundaries were applied as for the dual substrate incubations, i.e. an inhibition of at least 20% was considered relevant. Finally, activities in the intestinal microsomes were compared to the activities observed in hepatic microsomes. Eadie-Hofstee plots were derived as described for hepatic microsomes and profiles were compared. Activity ratios (V_max___*intestine*_/V_max___*liver*_) were calculated and used to assess enzyme involvement.

### Statistical analysis

Stepwise linear regression, correlation analyses, and independent sample t-tests were performed with the SPSS® 24 software (IBM, New York, USA). Sex differences in hepatic protein abundances, K_m_, and V_max_ values, were analyzed by an independent sample t-test. Normality was checked using the Shapiro Wilk test. If the Shapiro Wilk test statistic was significant, log transformation was used to calculate the t-statistic. Equality of variances was checked using the Levene’s test for equality of variances. The appropriate test statistic was used depending on the equality of variances.

### Ethical approval

 All procedures followed were in accordance to the ethical standards of the ethical committee of the Faculties of Veterinary Medicine and Bioscience Engineering of Ghent University (approval EC2015_213).

## Results

### Hepatic and intestinal CYP450 quantitative analysis

**Figure 1:**
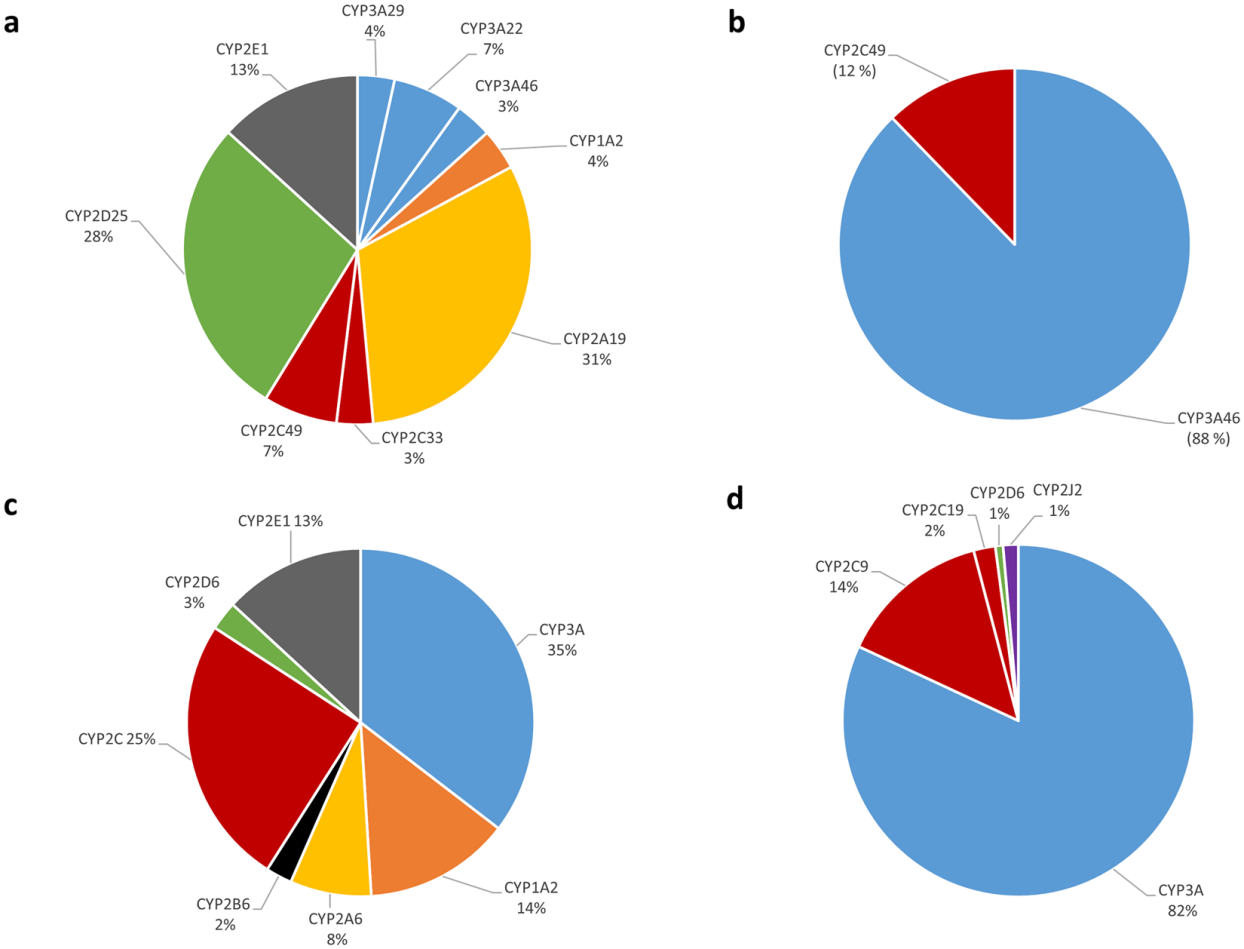
Relative amounts of hepatic (**a**) and duodenal (**b**) CYP450 proteins in conventional pigs and humans (**c**,**d** respectively) Total detected hepatic CYP450 was 365.3 ± 27.41 pmol/mg protein (n = 16; age 12 weeks, 8 males and 8 females), total detected duodenal CYP450 was 3.44 ± 2.42 pmol/mg protein (n = 3, pool of 16 pigs; 8 males and 8 females). Data for human CYP450 pie charts are derived from^43,76,87,88^.

The entry information, which provides the associated accession number(s), of the detected and quantified enzymes, are shown in Supplementary Table 1. The relative quantity of each enzyme is shown in Fig. 1, along with the relative quantity of the human isoforms. Mean amount of hepatic drug metabolizing CYP450 enzymes was 365.3 ± 27.41 pmol/mg protein, and for duodenal CYP450 3.44 ± 2.42 pmol/mg protein. In the jejunum and ileum, CYP450 were below the UPLC-HRMS limits of detection. The identified enzymes in the duodenum belong to the CYP2C and CYP3A subfamilies. No enzymes of other subfamilies, i.e. CYP1A, CYP2A, CYP2D and CYP2E were observed. No evidence for sex differences were noted for hepatic enzymes (minimum p-value = 0.309 for CYP3A29).

### Hepatic and intestinal CYP450 activity and inhibition

#### Hepatic and intestinal CYP450 activities

Mean values for Michaelis-Menten parameters of hepatic CYP450 of 8 male and 8 female conventional pigs, 12 weeks of age are shown in Table 2. Michaelis-Menten plots can be found in the Supplementary Files. No evidence for sex differences was observed in K_m_ and V_max_ (minimum p-value for K_m_ = 0.115 (TB) and for V_max_ p = 0.192 (DXM)), which is reflected in the CYP450 quantities. Consequently, the parameters provided in Table 2 represent the combined means for male and female pigs.

**Table 2:** Michaelis-Menten and Hill parameters for porcine hepatic CYP450 enzymes (8 males, 8 females, 12 weeks of age, each time 3 replicates).

	Porcine K_m_ (µM)	Porcine V_max_ (pmol/min/mg protein)	Porcine Hill coefficient	Human K_m_ (µM)	Human V_max_ (pmol/min/mg protein)	references
PH	20.0 (14.88)	1404 (403.6)	—	10–50	241–2173	^43, 81, 82^
CM	1.3 (0.47)	303 (119.2)	—	0.5–2	259–1275	^7, 10, 37, 43, 92^
MDZ	15.3 (4.4)	1848 (637)	1.23 (0.089)	3–9	190–4380	^43, 48, 50, 92, 93^
TB^**a**^	*2079* (*1821)*	*269* (*182*.*1*)	—	60–400	66–434	^15, 43, 76, 92, 94^
DXM	6.7 (5.70)	1592 (480.7)	—	2.2–8.5	18–233	^43, 86– 88, 92^
CZ	53.6 (18.39)	764 (259.1)	—	39–152	575–2301	^10, 21, 43, 45, 77, 78, 92, 95, 96^

Results are expressed as mean (and standard deviation). *V*_max_, maximal reaction rate; K_*m*_, Michaelis-Menten constant; PH, phenacetin; CM, coumarin; MDZ, midazolam; TB, tolbutamide; DXM, dextromethorphan; CZ, chlorzoxazone. ^a^Fitted coefficients for this compound should be interpreted with care as the reaction rate did not reach saturation.

**Figure 2:**
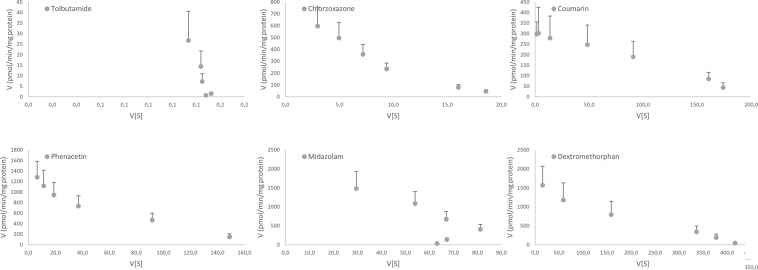
Eadie-Hofstee plots of CYP450 activity for tolbutamide, chlorzoxazone, coumarin, phenacetin, midazolam, and dextromethorphan, in porcine liver (n = 16, 8 males, 8 females, 12 weeks of age).

A hyperbolic Eadie-Hofstee plot for MDZ (CYP3A) was observed (Fig. 2), indicative for auto-activation or Hill kinetics^60^. The Eadie-Hofstee plots for CZ, PH, and TB indicate deviation from standard Michaelis-Menten kinetics. Chlorzoxazone and PH are biphasic, while TB does not saturate in the range of TB concentrations tested. A Michaelis-Menten equation with two contributing enzymes was fit to the CZ and PH data, but the estimates obtained were inaccurate and not significant. However, a fit to a single enzyme kinetic equation resulted in an adequate fit (Supplementary Fig. 1). For TB, however, the obtained parameters should be interpreted with care as they are estimated without observing saturation due to limitations in the TB solubility.

**Figure 3:**
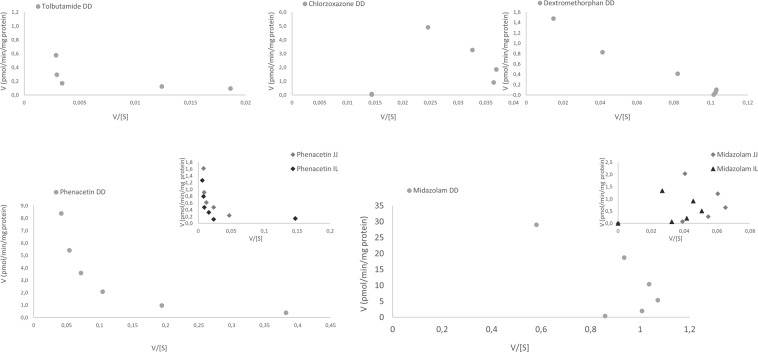
Eadie-Hofstee plots of CYP450 activity for tolbutamide, chlorzoxazone, and dextromethorphan in porcine duodenum (**DD**), and of phenacetin, midazolam in duodenum, jejunum (JJ) and ileum (IL).

For the intestinal CYP450, a gradual decrease in V_max_ was observed with duodenum > jejunum > ileum. No CM-hydroxylase activity could be detected in the intestine and only MDZ-hydroxylase and PH-O-deethylase activity were observed over all three segments (Table 3). Duodenal biotransformation of CZ, DXM, and MDZ (Fig. 3) displayed Hill kinetics and was consequently fitted against the Hill function.

**Table 3:** Michaelis-Menten and Hill parameters for porcine intestinal CYP450 enzymes (n = 3 replicates of a pool of 16 pigs, 8 males and 8 females, 12 weeks of age).

	Duodenum			Jejunum			Ileum		
	V_max_ (pmol/min/mg protein)	K_m_ (µM)	a	V_max_ (pmol/min/mg protein)	K_m_ (µM)	a	V_max_ (pmol/min/mg protein)	K_m_ (µM)	a
PH	14.1 (2.14)	143.5 (41.04)	na	2.97 (1.221)	181.7 (129.74)	na	2.41 (0.684)	188.6 (91.66)	na
CM	Nd	nd	nd	nd	nd	nd	nd	nd	nd
MDZ	39.5 (1.47)	22.1 (1.59)	1.26 (0.048)	2.71 (1.411)	23.1 (1.15)	1.41 (0.043)	1.57 (0.025)	16.2 (0.040)	1.56 (0.45)
TB	4.68 (3.383)	1407.5 (1248)	na	nd	nd	nd	nd	nd	nd
DXM	2.16 (0.186)	31.5 (8.13)	0.81 (3.873)	nd	nd	nd	nd	nd	nd
CZ	7.64 (0.458)	125.6 (13.18)	1.25 (0.058)	nd	nd	nd	nd	nd	nd

Results are expressed as mean (and standard deviation).

V_max_, maximal reaction rate; K_m_, Michaelis-Menten constant; a, Hill coefficient; PH, phenacetin; CM, coumarin; MDZ, midazolam; TB, tolbutamide; DXM, dextromethorphan; CZ, chlorzoxazone; na, not applicable; nd, not detected.

Intestinal CYP3A and CYP2C activity were much lower as would be expected from enzyme quantity ratios in hepatic versus intestinal microsomes. Ratios of CYP3A and CYP2C enzymes (hepatic/duodenal) were 3 and 10 respectively. However, V_max_ of MDZ-hydroxylation and TB-hydroxylation in intestinal microsomes were only 2% of the hepatic V_max_ values.

#### Inhibition of hepatic CYP450 by substrates and inhibitors

Results of the incubation with substrate-inhibitor and dual substrates are presented in Tables 4 and 5. In general, TB, CM and DXM had only a limited influence on the biotransformation of other substrates (inhibition <20%). In contrast, MDZ decreased almost every other activity except for CZ, which showed an increased rate. Furthermore, CZ and PH reduced the CM-hydroxylation, while CM did not decrease the CZ-hydroxylation and PH-O-deethylation to the same extent, even though CM has a much lower affinity.

**Table 4:** Influence of inhibitors at concentrations around their K_i_ values on different CYP450 enzyme activities in porcine hepatic microsomes. Results are presented as ratio of residual activity (reaction rate of substrate + inhibitor versus substrate only (control)). Value represents the mean of 3 replicate measurements (and standard deviation).

Subfamily	Inhibitor	CYP2C	CYP2E	CYP2A	CYP3A	CYP1A	CYP2D
		TB	CZ	CM	MDZ	PH	DXM
CYP2C	Sulphaphenazole	0.98 (0.135)	1.03 (0.061)	1.05 (0.023)	1.20 (0.050)	1.09 (0.030)	0.97 (0.080)
CYP1A/2A	α-naphthoflavone	1.13 (0.099)	1.06 (0.057)	0.94 (0.131)	1.08 (0.048)	0.76 (0.011)	1.02 (0.143)
CYP2D	Quinidine	0.97 (0.052)	1.07(0.044)	1.06 (0.072)	1.26 (0.075)	1.15 (0.016)	0.98 (0.080)
CYP3A	Ketoconazole	0.68 (0.107)	0.80 (0.011)	0.82 (0.073)	0.22 (0.007)	1.08 (0.066)	0.96 (0.100)
CYP2E	Diethyldithiocarbamate	0.87 (0.046)	0.87 (0.003)-	0.74 (0.079)	1.15 (0.074)	1.04 (0.010)	0.93 (0.084)
CYP2A	8-methoxypsoralen	1.01 (0.078)	0.72 (0.044)	0.13 (0.007)	1.29 (0.060)	0.68 (0.026)	0.95 (0.112)

TB, tolbutamide; CZ, chlorzoxazone; CM, coumarin; PH, phenacetin; MDZ, midazolam; DXM, dextromethorphan.

**Table 5:** Dual substrate incubations in porcine hepatic microsomes.

	TB	CZ	CM	PH	MDZ	DXM
TB	—	0.97 (0.026)	0.92 (0.021)	0.88 (0.024)	1.01 (0.031)	0.97 (0.043)
CZ	0.87 (0.014)	—	0.57 (0.012)	0.59 (0.006)	0.98 (0.053)	0.93 (0.073)
CM	0.89 (0.043)	0.86 (0.007)	—	0.83 (0.020)	0.92 (0.038)	0.99 (0.072)
PH	0.88 (0.033)	0.73 (0.041)	0.55 (0.027)	—	0.93 (0.032	0.95 (0.106)
MDZ	0.55 (0.041)	1.23 (0.019)	0.62 (0.027)	0.71 (0.011)	—	0.50 (0.034)
DXM	0.82 (0.153)	0.95 (0.050)	0.86 (0.106)	0.82 (0.082)	0.84 (0.115)	—

Results are presented as the ratio of the reaction rate of dual substrates versus single substrate incubations. The means are presented of 3 replicate measurements (and standard deviation).

TB, tolbutamide; CZ, chlorzoxazone; CM, coumarin; PH, phenacetin; MDZ, midazolam; DXM, dextromethorphan.

Sulphaphenazole and QND did not inhibit any of the investigated reactions, even if the concentration was increased to 10 times the concentration described in the materials and methods section. α-Naphthoflavone did not inhibit any reaction at a concentration around the K_i_ value for the human CYP1A2 enzyme (0.1 µM). However, when the concentration was increased to 1 µM, PH-O-deethylation (CYP1A2) activity decreased to 76% of the control value (Table 4). No other reactions were significantly inhibited by α-NFV at this concentration (maximal inhibition of 4% for CM).

At a concentration of 20 µM, DDC decreased the TB, CZ and CM hydroxylation. When the concentration was increased, all reactions were decreased by >40%. This indicates that DDC has no selectivity for the porcine CYP2E1 enzyme and thus cannot be used to draw conclusions about CYP2E1 enzyme involvement.

Ketoconazole and 8-MPS were found to be potent inhibitors of MDZ and CM-hydroxylation, respectively. However, KET could also inhibit TB-hydroxylation and 8-MPS inhibited CZ-hydroxylation and PH-O-deethylation, suggesting that either the inhibitors are not selective for CYP3A and CYP2A respectively or that the substrates are metabolized by CYP3A (TB) or CYP2A (CZ, PH). Notable is the apparent increase of MDZ-hydroxylation observed with all inhibitors, except KET.

### Selectivity assessment

**Figure 4:**
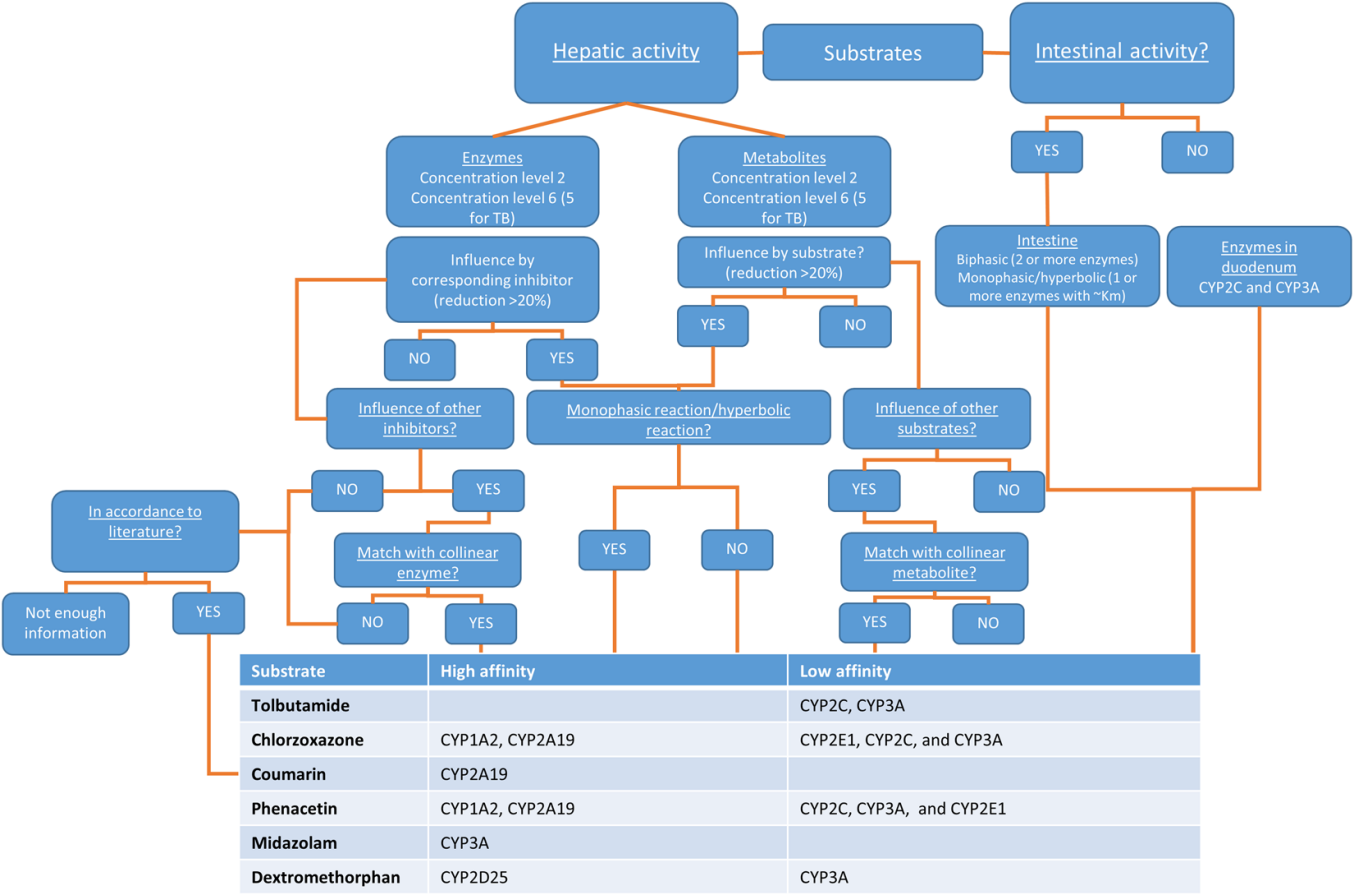
General decision tree for enzyme involvement. No distinction is made between enzymes belonging to the same subfamily. For a specific flow scheme for each substrate, the reader is referred to the Supplementary Data.

Eadie-Hofstee plots of hepatic and intestinal CYP450 activities are displayed in Figs 2 and 3. Correlation matrices between activity-enzyme and dual substrates can be found in the Supplementary Tables 2 and 3. Of note are the gradual increase or decrease in correlation coefficients as a function of concentration. Enzymes and metabolites selected by regression analysis can be found in Table 6. A general decision tree with associated outcome is given in Fig. 4. Detailed decision trees for each individual substrate are given in Supplementary Figs 3–8.

**Table 6:** Selected enzymes and metabolites by regression analysis.

Substrate	TB		CZ		CM		PH		MDZ		DXM	
Conc Level^a^	2	5	2	6	2	6	2	6	2	6	2	6
Enzymes	CYP2C49	CYP2C49 CYP3A46	CYP3A46 *CYP1A2*	CYP2E1 CYP2C49 *CYP1A2 CYP3A*	CYP2A19	CYP2A19	CYP2A19	CYP3A46 *CYP1A2*	CYP3A22	CYP3A46 CYP2C49 *CYP1A2*	CYP2C33 CYP3A46 *CYP1A2*	CYP3A46 *CYP1A2*
Metabolites	DEX	OH-CZ	PAR *OH-CM*	OH-TB OH-MDZ	PAR *OH-CZ*	PAR	OH-CM *OH-CZ*	OH-CM OH-CZ *OH-TB OH-MDZ*	PAR *OH-CZ OH-CM*	PAR OH-CZ *OH-TB OH-CM*	OH-TB	OH-MDZ

Enzymes and metabolites written in ‘italics’ are collinear variables with a tolerance value < 0.3. Criteria for inclusion and exclusion were a F-statistic <0.1 or >0.2, respectively.

TB, tolbutamide; CZ, chlorzoxazone; CM, coumarin; PH, phenacetin; MDZ, midazolam; DXM, dextromethorphan; DEX, dextrorphan; OH-CZ, 6-OH-chlorzoxazone; OH-CM, 5-hydroxy-coumarin; PAR, paracetamol; OH-TB, 9-OH-tolbutamide; OH-MDZ, 1-OH-midazolam.^*a*^Concentration levels are specified in Table 1.

## Discussion

To the best of our knowledge, this is the first study to quantify and characterize porcine intestinal CYP450 enzymes. Duodenal relative quantitative results are remarkably similar between pigs and men, with CYP3A constituting 88% and CYP2C 12% of the detected CYP450 enzymes in pigs vs. 82% CYP3A and 16% CYP2C in humans (Fig. 1)^65^. However, intestinal CYP450 activity is much lower in porcine microsomes, especially for CYP3A, which is 6–40 times lower^66,67^. In contrast to the V_max_ of MDZ-hydroxylation, the V_max_ for TB-hydroxylation (CYP2C) is similar to those observed in humans^67^, although direct comparison is difficult due to the different amounts of intestinal sample used for microsomes preparation. Hence no systematic comparison could be performed. In the current study, intestinal pieces of 4 grams were processed, while in previous studies^66,67^ mucosal scrapings of 1 foot sections or entire gut length were used. Likewise, the observed discrepancy for the CYP3A activity, can be attributed in part to the difference in intestinal microsomes preparation. Previously it has been shown that the elution of intestinal samples with EDTA resulted in the highest CYP450 activity when compared to the mucosal scraping method^68^. Furthermore, in the comparison between the EDTA elution method and the mincing method, it was shown that the latter resulted in higher enzymatic activities and more reproducible results^69^. Hence the mincing method was used in the current study.

The current study shows that the total detected amount of hepatic CYP450 enzymes is similar between pigs and humans (365 vs. 240–303 pmol/mg protein)^70,71^. Relative amounts of porcine hepatic CYP450 enzymes are in agreement with the results of Achour *et al*. (2011). They investigated the CYP450 quantity in two adult conventional pigs, with a maximal difference of 5% in relative quantity for CYP2E1 compared to the current study. No evidence for sex differences was found, which corresponds with the recent results of Millecam *et al*. (2018)^72^. However, compared to humans, differences in relative quantities are observed, with CYP2C, CYP1A2, and CYP3A being less abundant (10 vs. ~25%, 4 vs. 10–18%, and 14 vs. 32–38% in pigs vs. humans, respectively) and CYP2A, CYP2D, and CYP2E being more abundant (31 vs. ~7%, 28 vs. ~2%, and 13% vs. 8–17%, in pigs vs humans, respectively) (Fig. 1)^70,73,74^.

The 2.5 times lower quantity of porcine CYP2C enzymes seems to be reflected in the lower TB-hydroxylase activity compared to human^15^. However, it should be mentioned that due to the limited concentration range of TB, with no obvious observable saturation of the kinetics, it is recognized that the K_m_ and V_max_ estimates reported herein can be biased. Nevertheless, previous reported V_max_ values in porcine hepatic microsomes range from 62–440 pmol/mg protein/min^15,19,75^ which are similar to the values reported here and within the range reported for humans. The affinity, on the other hand, is approximately 5 to 10 times lower^76^. It should be mentioned that the K_m_ estimate of tolbutamide differs somewhat from previous experiments by Schelstraete *et al*. (2018)^59^. This can be attributed to the fact that for the K_m_ estimation in the latter reference, the highest concentration level was included. In addition, the microsomes originated from different animals as described in this study.

In contrast to the similar biotransformation, SFZ did not inhibit TB-hydroxylation, indicating some qualitative differences with the human orthologues^19^. Nevertheless, with respect to the selectivity, the CYP2C enzymes were most likely the major contributors to TB-hydroxylation, as they showed strong correlations with the activity. Furthermore, TB did not influence the biotransformation of other probes. However, KET could also inhibit the reaction moderately, a feature also seen in humans^64^. Therefore, in addition to the increasing correlations with MDZ-hydroxylation and CYP3A46, and biphasic intestinal metabolism, CYP3A enzymes probably contribute to a low affinity phase.

The V_max_ of the CZ-hydroxylation reaction was similar between porcine and human microsomes (764 vs. 575–3,100 pmol/min/mg protein)^15,77^. Remarkably the porcine and human CYP2E1 are almost equally abundant, representing 13% and 9% of drug-metabolizing CYP450 respectively^70^. However, the selectivity of chlorzoxazone for CYP2E1 has been questioned^43,47,52^. In humans, CYP1A2 and CYP3A also contribute to the reaction^78–80^, whereas in pigs recombinant CYP1A1, CYP2A19, and CYP2C33 are able to hydroxylate CZ^47^.

At low substrate concentrations, CYP3A46 and PAR were selected from the regression analysis. Nonetheless, KET could only marginally inhibit CZ-hydroxylation (20%) and α-NFV did not inhibit the reaction. In contrast, 8-MPS inhibited the reaction to about 28%. Furthermore, CM, PH, and CZ affected each other’s biotransformation significantly, leading to the conclusion that mainly CYP2A19 and CYP1A2 are responsible for CZ-biotransformation. Indeed, when combining the CYP450 amounts in microsomes with data from recombinantly expressed CYP450^47^, CYP2A19 and CYP1A2 account for 72% of CZ-hydroxylation at a 5 µM concentration.

At higher substrate concentrations, CYP2E1, in addition to CYP2C and CYP3A enzymes, most likely metabolize CZ. Even though no marked influence was seen on CZ-hydroxylation by TB or MDZ, the associated parallel increase in correlation coefficient between CZ-hydroxylation and CYP3A, CYP2C enzymes at one hand, and TB and MDZ on the other hand, in combination with duodenal activity, suggests their involvement. However, Wiercinska and Squires expressed recombinant porcine CYP3A, but were unable to detect CZ-hydroxylase activity from this enzyme^47^. Of note is that their cloned CYP3A differed 2 amino acids from the detected CYP3A29 in this study. Finally, at higher concentrations CYP2E1 was the variable explaining most of the variability in CZ-hydroxylation, suggesting its role in catalyzing the reaction.

Coumarin is almost exclusively used as CYP2A6 probe^43^. The CYP2A19 enzyme represents 31% of the hepatic CYP450 enzymes with a comparable V_max_ value for humans, depending on the reference (Table 2). Coumarin-hydroxylation is proposed to be specific in pigs^44^, indicating that porcine CYP2A19 is less efficient. The current study also shows that coumarin is selective for CYP2A19. However, CM-hydroxylation and PH-O-deethylation correlated significantly, indicating a shared enzyme. As a consequence of the strong inhibition by 8-MPS (87%) and weak inhibition of the CYP1A2 associated inhibitor α-NFV (5%), the monophasic Eadie-Hofstee plot, and the absence of intestinal biotransformation, CYP2A19 is likely the sole important contributor to CM-hydroxylation.

In contrast, PH, a typical human CYP1A2 probe, is most likely metabolized in part by CYP2A19. This is supported by the fact the V_max_ of the PH-deethylation is similar (V_max_ 1,404 vs 241–2173^81,82^, pig and human), although the amount of CYP1A2 is 4 times lower in pigs compared to humans suggesting higher efficiency of porcine CYP1A2 or involvement of additional enzymes. Indeed, as PH and CM could affect the other’s biotransformation rate, and 8-MPS inhibited PH-O-deethylation, CYP2A19 is likely involved. Hence, CYP2A19 seems to have broader substrate selectivity compared to human CYP2A6 as indicated by its capability of metabolizing PH and CZ. This apparent broader substrate selectivity of CYP2A19 is further supported by a report in which 1-ethoxyresorufin, another frequently used CYP1A2 substrate, was found to markedly affect coumarin-hydroxylation when co-incubated^83^. Finally, α-NFV was able to inhibit PH-O-deethylation (24%), indicating that CYP1A2 is involved in its biotransformation.

At higher concentrations, additional enzymes contribute to the PH-deethylation. The observation of a similar biphasic plot in hepatic and duodenal microsomes, suggests involvement of CYP2C and CYP3A enzymes, although in hepatic microsomes KET could not inhibit PH-O-deethylation at concentrations around the K_m_ value. Nevertheless, strong correlations (r = 0.80) were observed between PH-O-deethylation and MDZ-hydroxylation. In addition, CM- and CZ-hydroxylation explained most of the PH-O-deethylation variability. Although CZ is not very selective for CYP2E1, a parallel increase in correlation with this enzyme suggests CYP2E1 has low affinity for PH-deethylation, as observed in humans^84^.

The MDZ-hydroxylation rate is about 2 to 3 times lower compared to humans^48^, paralleled by the approximately 3 times lower amount of CYP3A observed in the current study. Our results indicate that MDZ is largely selective for porcine CYP3A. Similar Eadie-Hofstee plots in hepatic and intestinal microsomes indicate the involvement of the same enzymes. Remarkably, MDZ reduced all reactions except CZ-hydroxylation, although this results possibly from nonselective binding of MDZ or a metabolite thereof, as supported by the observation of a sigmoidal profile, a characteristic observed for nonselective binding of other weak bases^85^. In addition, all inhibitors, except KET, enhanced MDZ-hydroxylation, which can be hypothesized to result from an increase in free concentration.

It has been shown that rCYP3A22 had the highest intrinsic MDZ clearance, followed by rCYP3A29 and rCYP3A46^48^. CYP3A22 had the highest quantity amongst CYP3A enzymes, which is reflected by the significant correlations with MDZ biotransformation at all concentrations. No significant correlation was found for CYP3A29, although it has a high intrinsic clearance and is the second highest quantified CYP3A enzyme^48^. A possible explanation is a polymorphism in the CYP3A29 sequence, supported by the existence of a CYP3A sequence in the Uniprot database, differing in only two amino acids (Asn423->His, Lys458->Arg). Recently, this sequence was recombinantly expressed. Unfortunately no CYP3A substrate was measured^47^.

Dextromethorphan-O-demethylation was exceptionally high, being up to 7 times faster in pigs than in humans (1,593 vs. 18.0–233 pmol/min/mg protein)^86–88^, which reflects the 14 times higher quantity of CYP2D (28% vs. 2% in humans)^20,70,89,90^. However, porcine DXM-metabolism has been debated with CYP2B22 rather than CYP2D25 being responsible for DXM-O-demethylation^20,89,90^. Nevertheless, no CYP2B22 was observed in this study. Hence it is unlikely that CYP2B22 is the most important enzyme in DXM-O-demethylation.

Regression analysis selected CYP3A46, CYP2C33, OH-TB, and OH-MDZ as predictive variables. However, KET could not inhibit DXM-O-demethylation and only MDZ had a marked influence, on the DXM-biotransformation, although this probably resulted from nonselective binding. As a consequence, these enzymes are most likely not the major contributors to the DXM biotransformation. CYP2D25, however, also correlated significantly with the DXM-O-demethylation (r = 0.55–0.66). Although QND did not inhibit the reaction, in line with other reports^20,89^, the observed correlation in addition to the high biotransformation rate and linear Eadie-Hofstee plot, which is indicative for single enzyme involvement at the used concentrations, suppose that CYP2D25 is most likely responsible for DXM-biotransformation.

## Conclusions

Tolbutamide, CM, MDZ, and DXM selectivity is similar between pigs and humans. This is an important feature for a potential preclinical animal species, as most of the currently available drugs are metabolized by CYP2C, CYP3A, and CYP2D. Moreover, being capable of producing the same metabolites at more or less equal rates is crucial in the safety and toxicological assessment of new drug candidates, as not only the parent molecule but also the metabolites can give rise to safety concerns. In contrast, CZ and PH are metabolized in part by CYP2A19. In humans, CZ is not metabolized by CYP2A6 and PH only to a limited extend^84,91^. This may be due to the higher amount and broader substrate-selectivity of CYP2A19. In addition, SFZ and QND showed no inhibition towards CYP2C and CYP2D25 respectively, indicating some differences in binding characteristics compared to human CYP450 orthologues. To conclude, the results presented herein support the use of the pig as an appropriate animal species for drug metabolism studies, based on the similarities in selectivity- and quantity- normalized activities, especially for the most important CYP3A subfamily. The current study provided an important step towards a consistent and systematic validation of the pig as a model for human drug metabolism.

## Supplementary information

Supplementary file

Supplementary Dataset 1

## Data Availability

All data analyzed during this study are included in the published article and its Supplementary Information Files. The raw proteomics data will be made available to one of the public repositories mentioned in the Editorial and Publishing policies.
