# Supplementary material for: Characterization of Porcine Hepatic and Intestinal Drug Metabolizing CYP450: Comparison with Human Orthologues from A Quantitative, Activity and Selectivity Perspective
Source: Sci Rep. 2019 Jun 25;9:9233. doi: 10.1038/s41598-019-45212-0 (PMC6592956; doi:10.1038/s41598-019-45212-0)
Supplement: Supplementary file 1 — Supplementary file [file 41598_2019_45212_MOESM1_ESM.docx]

**Characterization of porcine hepatic and intestinal Drug metabolizing CYP450: comparison with human orthologues from a quantitative, activity and selectivity perspective**

**Supplementary data**

Wim Schelstraete, Laura De Clerck, Elisabeth Govaert^,^ Joske Millecam, Mathias Devreese, Dieter Deforce, Jan Van Bocxlaer and Siska Croubels

Supplementary Table 1. Entry information of, detected CYP450 enzymes and mean concentrations (n=16 for liver; n=3 replicates for intestine duodenal section (pool of 16 pigs)). SEM, standard error of the mean; CV %, coefficient of variation.

| **Liver (n=16)** | | | | |
| --- | --- | --- | --- | --- |
| **Entry n°** | **CYP Enzyme** | **Mean (pmol/mg protein)** | **SEM** | **CV %** |
| D0G6S0_PIG | CYP51A1 | 6,26 | 1,76 | 28.1 |
| CP4AP_PIG | CYP4A21 | 21,76 | 6,62 | 30.4 |
| CP3AT_PIG | CYP3A29 | 12,57 | 3,52 | 28.0 |
| F1SC62_PIG | CYP2C49 | 25,08 | 18,57 | 74.1 |
| A0A0H4IV15_PIG | CYP3A22 | 23,67 | 10,54 | 44.5 |
| F1SJ26_PIG | CYP1A2 | 13,97 | 4,11 | 29.4 |
| CP2DP_PIG | CYP2D25 | 102,1 | 34,32 | 33.6 |
| A7KZR2_PIG | CYP3A46 | 12,43 | 6,26 | 50.4 |
| CP2E1_PIG | CYP2E1 | 48,41 | 15,67 | 32.4 |
| A0A0H4J4A5_PIG | CYP2C33 | 12,33 | 4,97 | 40.3 |
| A7KZR6_PIG | CYP27A1 | 9,53 | 5,19 | 54.4 |
| Q8SQ68_PIG | CYP2A19 | 114,7 | 50,17 | 43.7 |
| **Duodenum (n=3)** | | | | |
| A0A0H4IRA9_PIG | CYP20A1 | 0.39 | 0.043 | 19.0 |
| A0A0H4J4A1_PIG | CYP2C49 | 0.17 | 0.060 | 61.6 |
| CP3AT_PIG | CYP3A29 | 1.2 | 0.54 | 76.8 |

Supplementary Table 2. Significant Pearson’s correlation coefficients between hepatic CYP450 abundancies and probe activities in conventional pigs. Numbers below the enzyme column indicate the specific isoform.

|  | **TB** | | **CZ** | | | | | **CM** | **PH** | | **MDZ** | | | **DXM** | | | | | |
| --- | --- | --- | --- | --- | --- | --- | --- | --- | --- | --- | --- | --- | --- | --- | --- | --- | --- | --- | --- |
|  | **CYP2C** | | **CYP2C** | | **CYP3A** | | **CYP2E** | **CYP2A** | **CYP3A** | **CYP2A** | **CYP3A** | | **CYP1A** | **CYP3A** | | **CYP2D** | **CYP1A** | **CYP2E** | **CYP2C** |
|  | **49** | **33** | **49** | **33** | **46** | **22** | **1** | **19** | **46** | **19** | **22** | **46** | **2** | **29** | **46** | **25** | **2** | **1** | **33** |
| **1^a^** | 0.64* | 0.53 | - | - | - | - | - | - | - | - | 0.54 | - | - | - | 0.59 | - | - | 0.55 | 0.59 |
| **2** | 0.65* | 0.55 | - | - | - | - | - | - | - | 0.51 | 0.62 | - | - | 0.52 | 0.65* | 0.55 | - | 0.57 | 0.65* |
| **3** | 0.68* | 0.62* | - | - | 0.61 | - | - | 0.62 | - | 0.57 | 0.69* | - | - | 0.56 | 0.73* | 0.57 | 0.54 | 0.60 | 0.65* |
| **4** | 0.71* | 0.62* | 0.58 | 0.53 | - | - | 0.58 | 0.66* | - | - | 0.71* | - | - | 0.58 | 0.78* | 0.63* | 0.56 | 0.56 | 0.70* |
| **5** | 0.69* | 0.62* | 0.56 | 0.56 | - | 0.55 | 0.59 | 0.67* | - | - | 0.72* | 0.57 | 0.51 | 0.58 | 0.75* | 0.63* | 0.55 | 0.52 | 0.70* |
| **6** | 0.72* | 0.61 | 0.53 | 0.54 | - | 0.54 | 0.60 | 0.66* | 0.51 | - | 0.65* | 0.68* | 0.56 | 0.59 | 0.74* | 0.66* | 0.54 | 0.54 | 0.67* |

Only significant correlations are given (p-value <0.05).*P-value <0.01. ^a^ Concentrations are given as levels from 1-6 for clarity purposes. Concentrations corresponding to each level can be found in table 1. TB, tolbutamide: CZ, chlorzoxazone; CM, coumarin; PH, phenacetin; MDZ, midazolam; DXM, dextromethorphan;

Supplementary Table 3. Significant Pearson’s correlations coefficients between two probe activities in conventional pigs.

|  | **TB-CZ** | **TB-MDZ** | **TB-DXM** | **CZ-CM** | **CZ-PH** | **CZ-MDZ** | **CZ-DXM** | **CM-PH** | **PH-MDZ** | **PH-DXM** | **MDZ-DXM** |
| --- | --- | --- | --- | --- | --- | --- | --- | --- | --- | --- | --- |
| **1^a^** | - | - | 0.57 | 0.84* | 0.95* | 0.57 | - | 0.87* | - | - | - |
| **2** | - | - | 0.60 | 0.84* | 0.96* | 0.55 | - | 0.89* | 0.55 | - | - |
| **3** | 0.53 | 0.60 | 0.58 | 0.55 | 0.81* | 0.66* | 0.65* | 0.83* | 0.56 | - | 0.50 |
| **4** | 0.82* | 0.68* | 0.55 | - | 0.65* | 0.79* | 0.64* | 0.82* | 0.53 | - | 0.47 |
| **5** | 0.87* | 0.68* | 0.52 | - | 0.57 | 0.85* | 0.58 | 0.69* | 0.60 | - | 0.55 |
| **6** | 0.84* | 0.61 | - | - | 0.65* | 0.80* | 0.51 | 0.66* | 0.80* | 0.54 | 0.61 |

Only significant correlations are given (p-value <0.05).*P-value <0.01. TB, tolbutamide: CZ, chlorzoxazone; MDZ, midazolam; DXM, dextromethorphan; CM, coumarin; PH, phenacetin. ^a^Numbers in de left column indicate the concentration levels tested. Corresponding concentrations can be found in table 1.

Supplementary Table 4. Human K_m_ and K_I_ values for the used substrates and inhibitor in the current study

| Substrate | Km (µM) | reference |
| --- | --- | --- |
| Phenacetin | 10-50 | (Yuan *et al.*, 2002) and references herein |
| Coumarin | 0.5-2 |  |
| Tolbutamide | 60-400 |  |
| Dextromethorphan | 2.2-8.5 |  |
| Midazolam | 3-5 |  |
| Chlorzoxazone | 40 |  |
| Inhibitor | Ki (µM) | reference |
| α-Naphthoflavone | 0.013 | (Cyrus *et al.*, 2011) and references herein |
| 8-Methoxypsoralen | 0.1-1.5 |  |
| Sulphafenazole | 0.12-0.7 |  |
| Quinidin | 0.03-0.4 |  |
| Ketoconazol | 0.004-0.13 |  |
| Diethyldithiocarbamate | 12.2-38 | (Baranovà *et al.*, 2005; Pratt-hyatt *et al.*, 2010) |


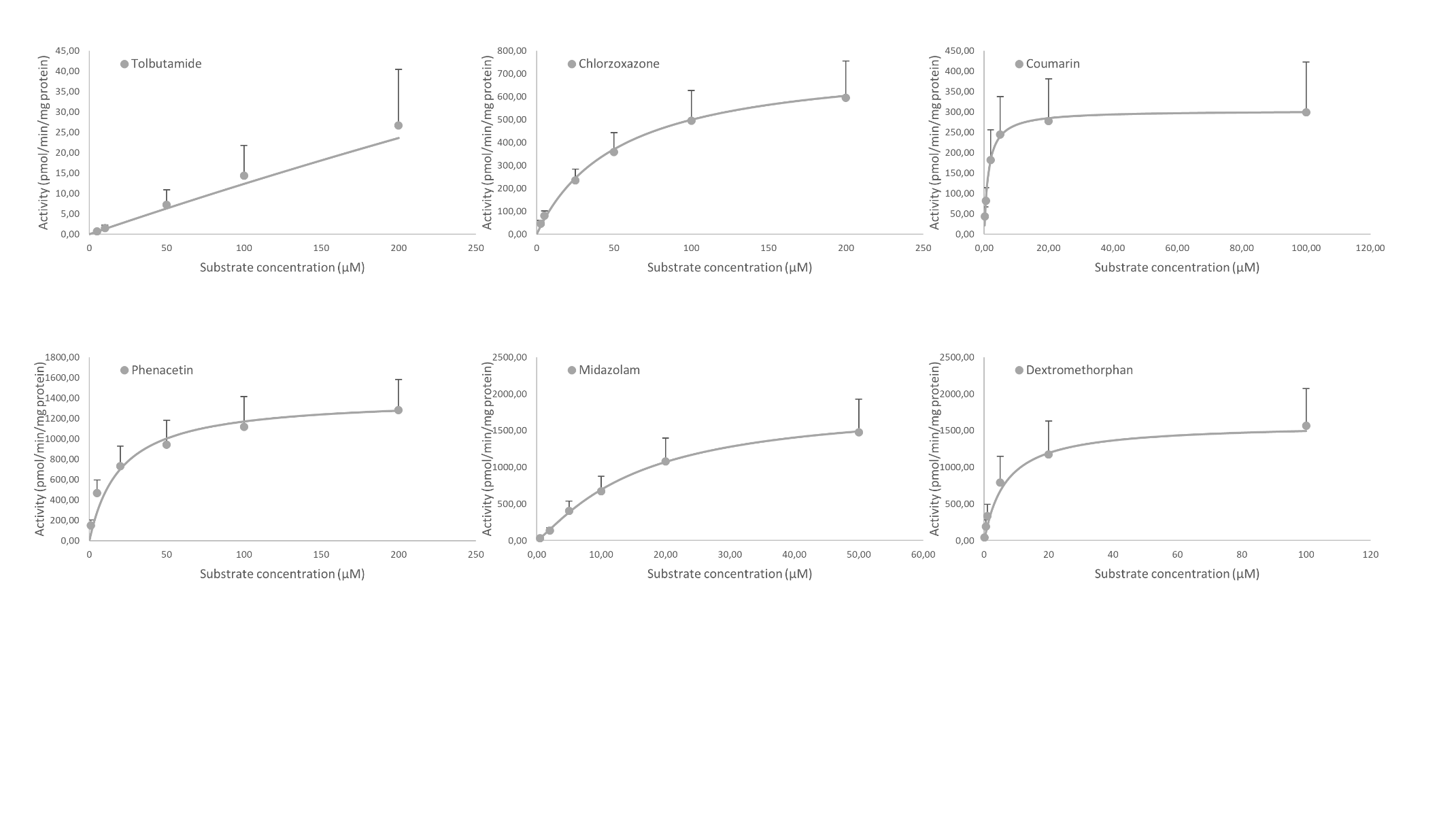


*Supplementary Figure 1 Michaelis Menten plots of CYP450 activity for tolbutamide (a), coumarin, phenacetin, midazolam, dextromethorphan (b), and chlorzoxazone (c) in porcine liver (n= 16, 8 males, 8 females, 12 weeks of age).*


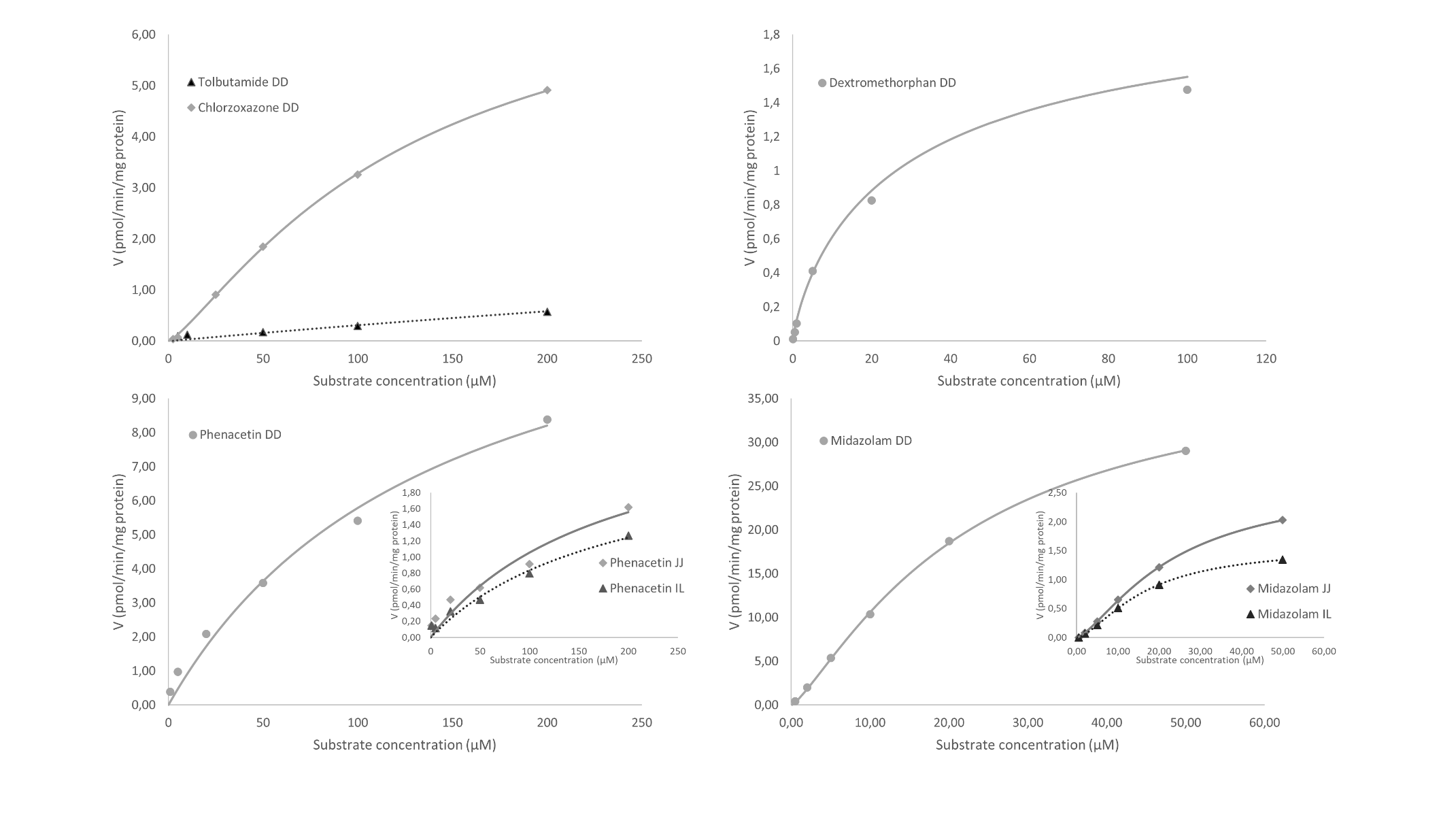


*Supplementary Figure 2 Michaelis Menten plots of CYP450 activity for tolbutamide and chlorzoxazone in porcine duodenum (a), dextromethorphan (b) and of phenacetin, midazolam in duodenum (DD), jejunum (JJ) and ileum (IL) (c and d respectively).*


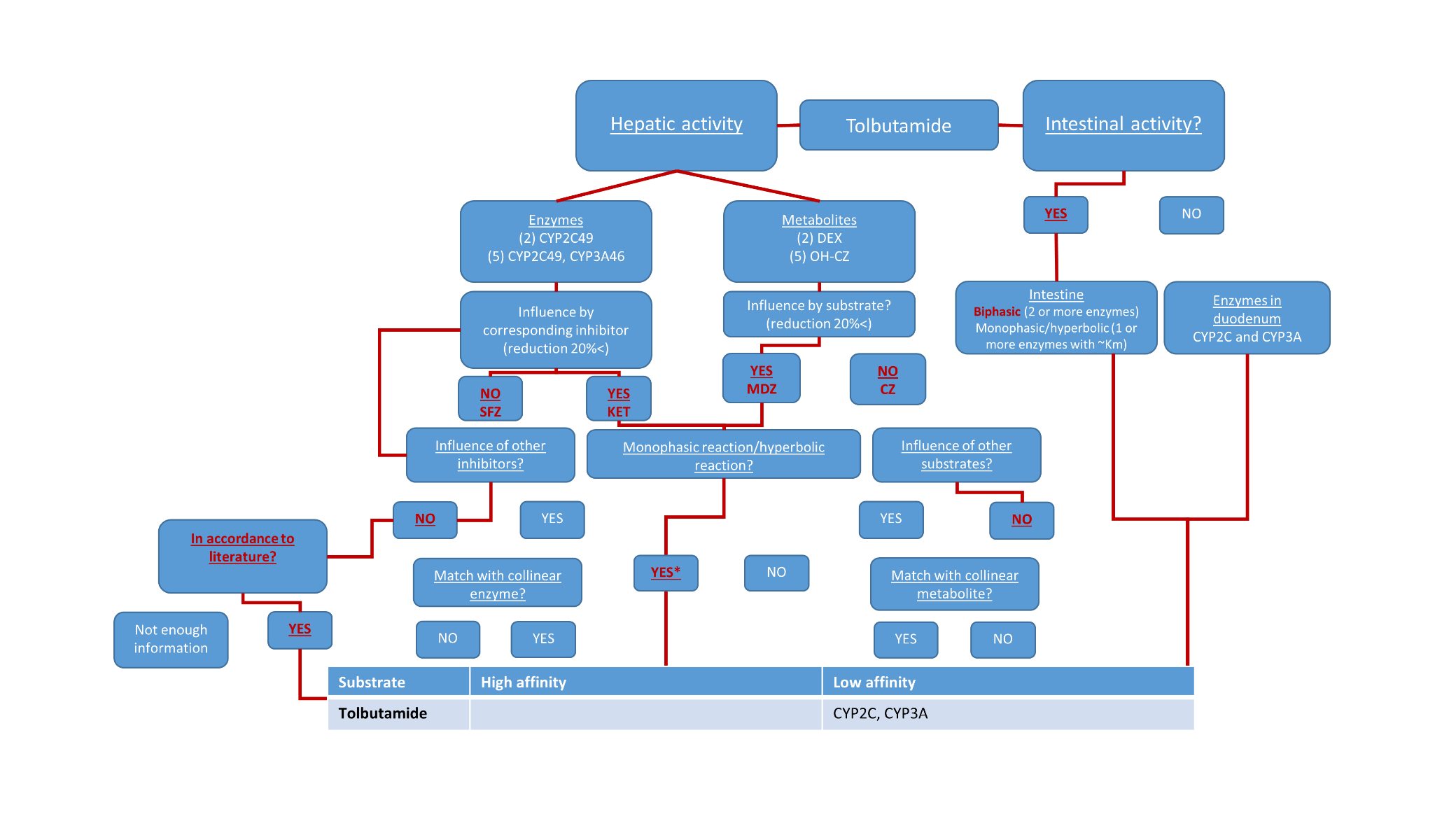
Supplementary Figure 3: decision tree for analysis of enzyme involvement in tolbutamide-hydroxylation. SFZ, sulphaphenazole; KET, ketoconazole; MDZ, midazolam; OH-CZ, hydroxy-chlorzoxazone; DEX, dextrorphan. *MDZ inhibited all reactions except chlorzoxazone-hydroxylation, possibly due to non-selective binding.


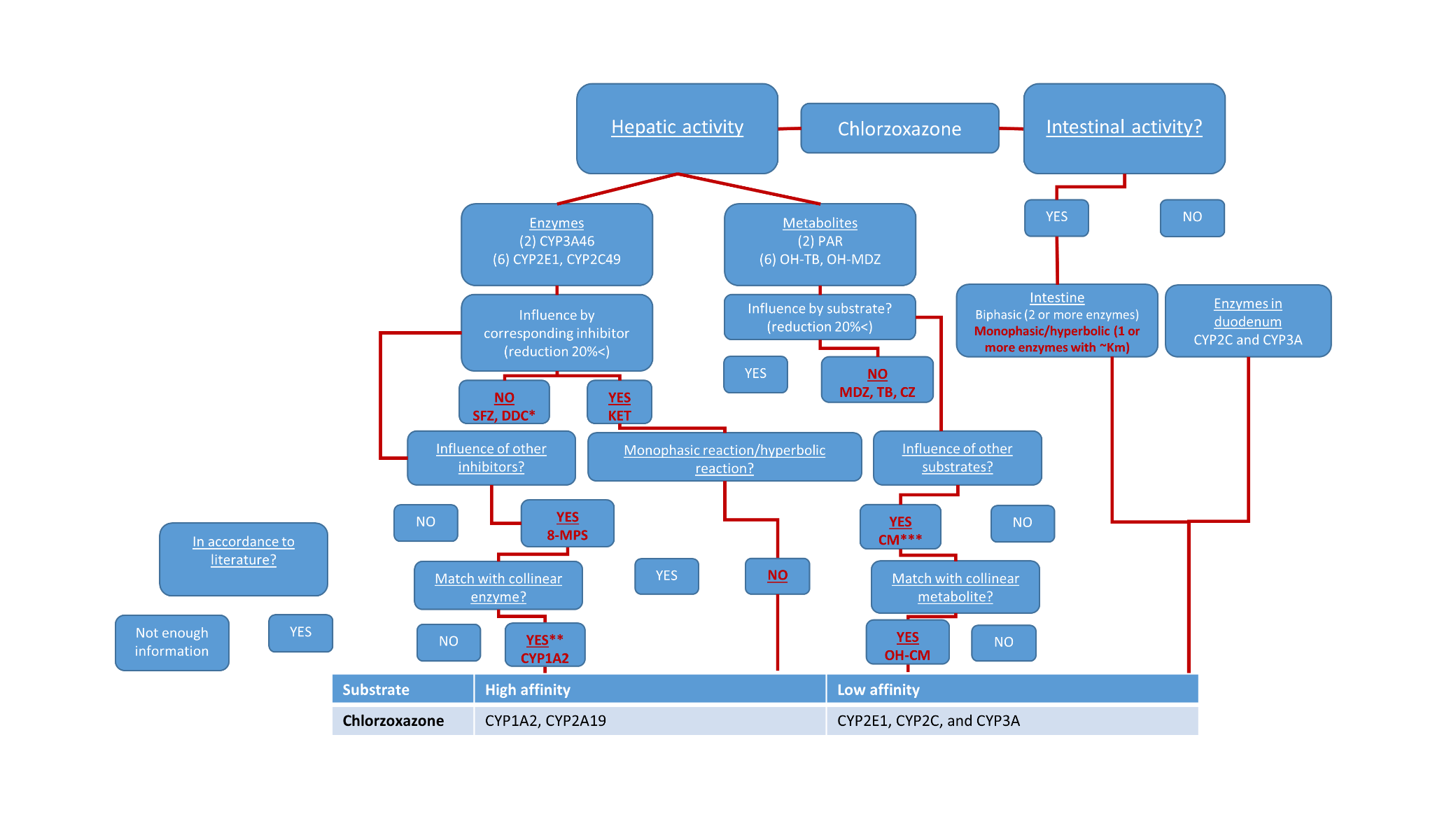
Supplementary Figure 4: decision tree for analysis of enzyme involvement in chlorzoxazone-hydroxylation. SFZ, sulphaphenazole; DDC, diethyldithiocarbamate; KET, ketoconazole; MDZ, midazolam; PAR, paracetamol; OH-TB, hydroxy-tolbutamide; OH-CZ, hydroxy-chlorzoxazone; TB, tolbutamide; CZ, chlorzoxazone; OH-CM, hydroxy-coumarin. *DDC was not selective for CYP2E1. **8-MPS is able to inhibit phenacetin-O-deethylation and also inhibits CYP1A2 in men. *** CZ and CM affected each other’s biotransformation (14% inhibition by of CZ by CM and 41% inhibition of CM by CZ)


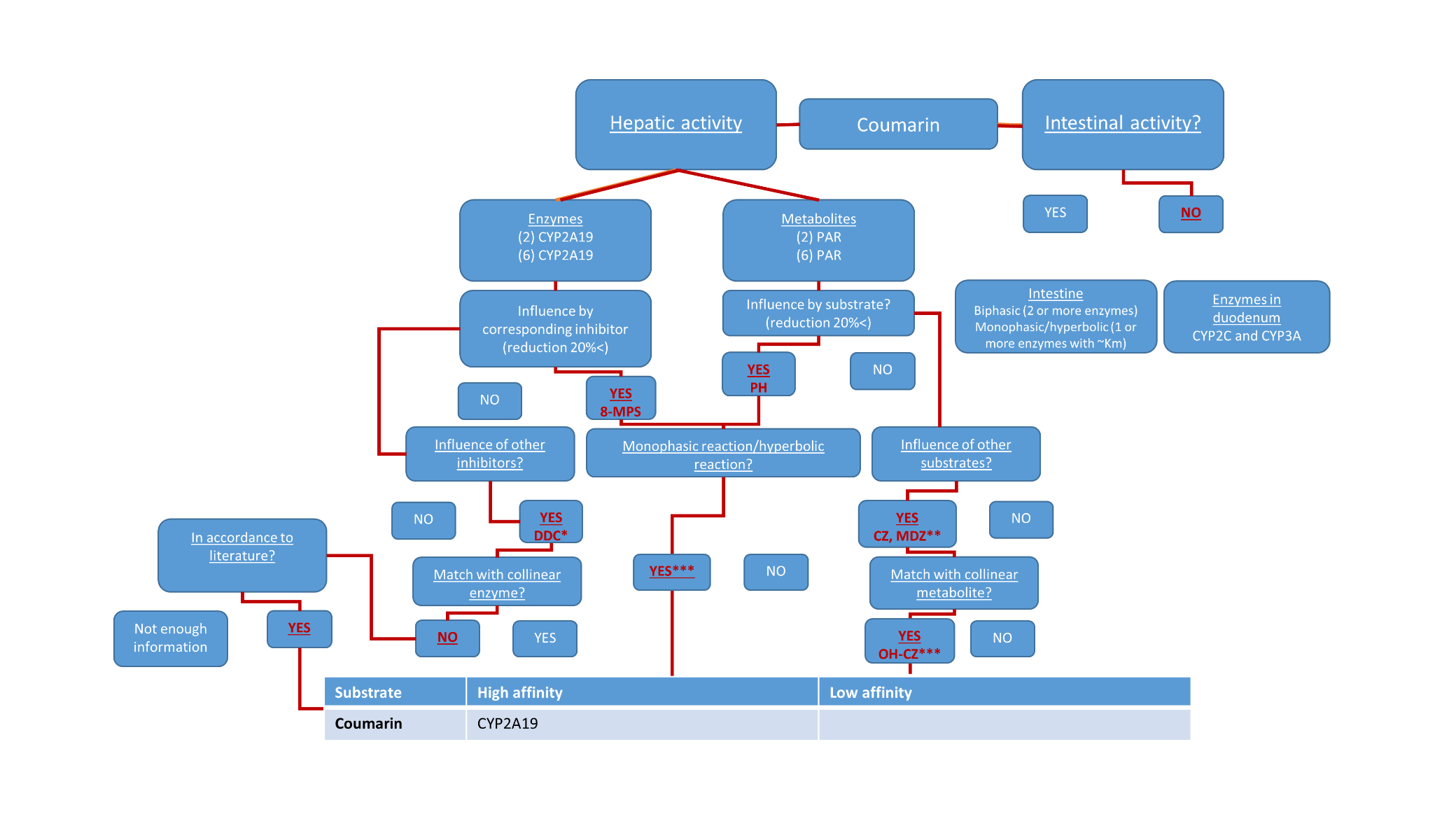
Supplementary Figure 5 decision tree for analysis of enzyme involvement in coumarin-hydroxylation. DDC, diethyldithiocarbamate; MDZ, midazolam; PAR, paracetamol; PH, phenacetin; OH-CZ, hydroxy-chlorzoxazone; CZ, chlorzoxazone;. *DDC inhibition was considered not selective. **MDZ inhibited all reactions except chlorzoxazone-hydroxylation, possibly due to non-selective binding. ***PH considered to be metabolised in part by CYP2A19.


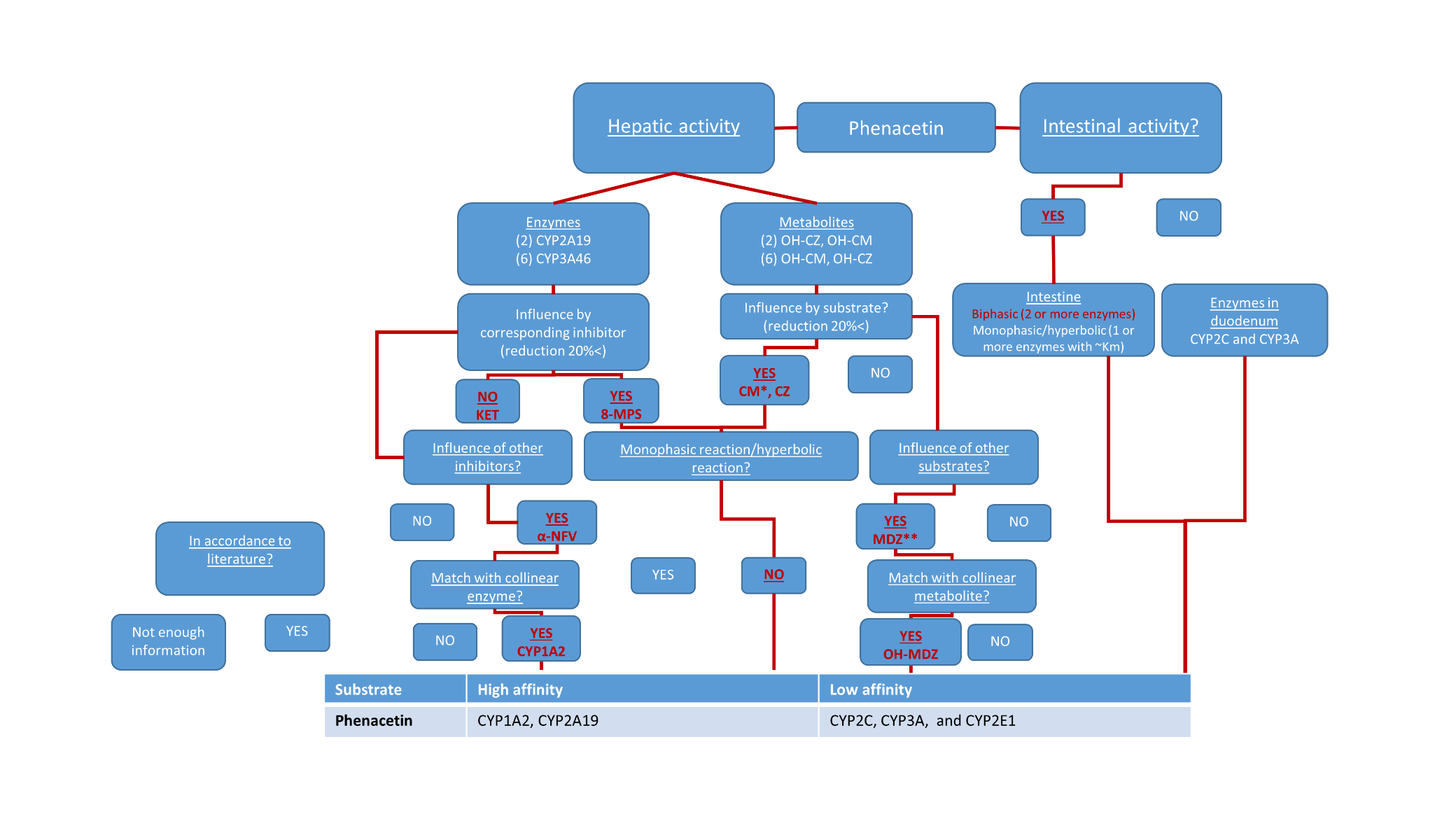
Supplementary Figure 6 decision tree for analysis of enzyme involvement in phenacetin-O-deethylation. KET, ketoconazole; 8-MPS, 8-methoxypsoralen; α-NFV, α-naphthoflavone; OH-CZ, hydroxy-chlorzoxazone; OH-CM, hydroxy-coumarin; CM, coumarin; CZ, chlorzoxazone; MDZ, midazolam. *CM inhibition of PH was 17%, while inhibition of PH on CM was 45%. Therefore, CM was considered to influence phenacetin-O-deethylation. **MDZ inhibited all reactions except chlorzoxazone-hydroxylation, possibly due to non-selective binding


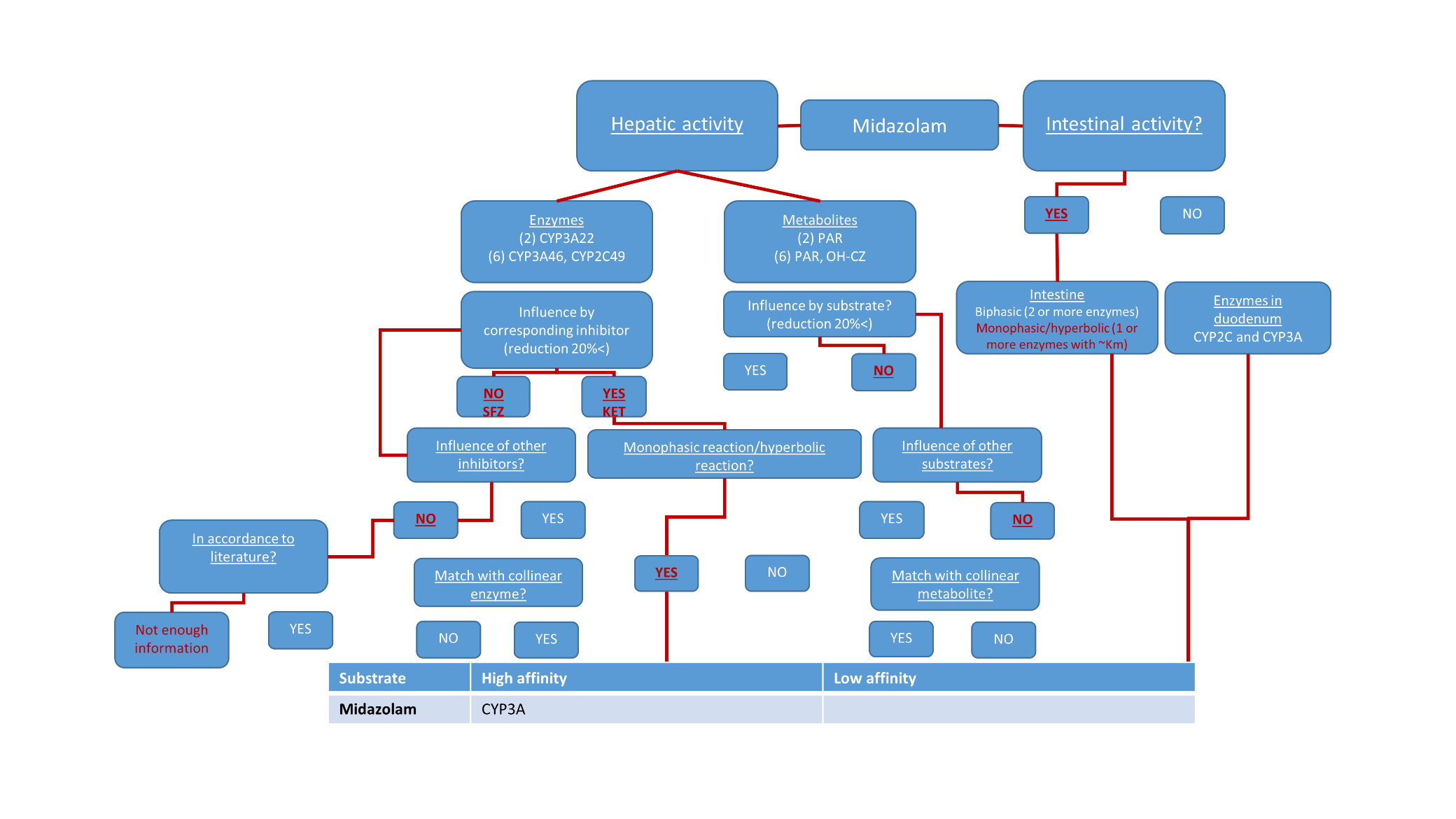
Supplementary Figure 7 decision tree for analysis of enzyme involvement in midazolam-hydroxylation. SFZ, sulphaphenazole; KET, ketoconazole; PAR, paracetamol, OH-CZ, hydroxy-chlorzoxazone.


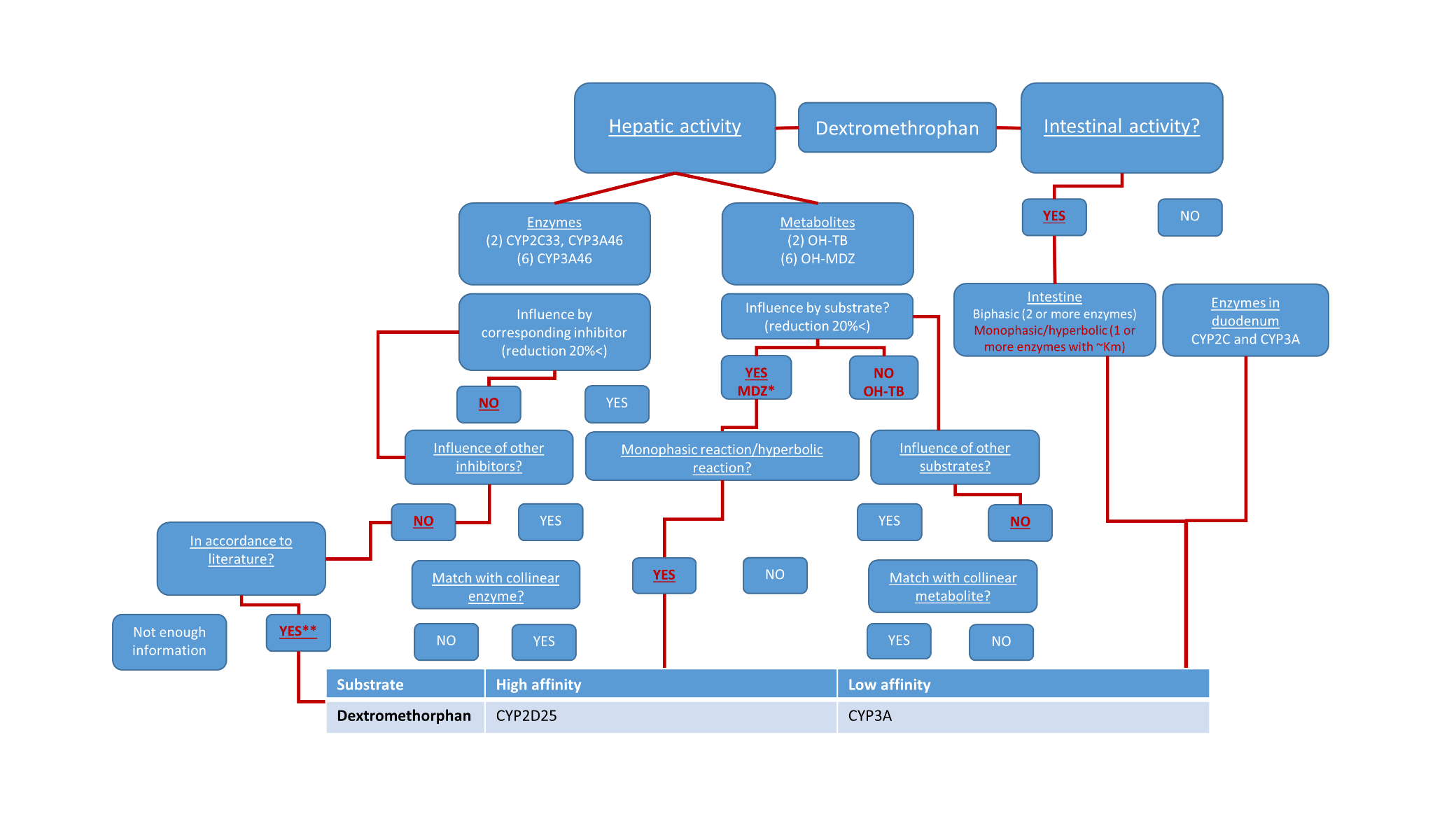
Supplementary Figure 8 decision tree for analysis of enzyme involvement in dextromethorphan-O-demethylation. OH-TB, hydroxy-tolbutamide; MDZ, midazolam. * MDZ inhibited DXM-O-demethylation by 50%, although KET could not inhibit the reaction, indicating that CYP3A or CYP2C enzymes only play a minor role in the DXM-O-demethylation reaction. **Quinidine has been shown not to inhibit porcine CYP2D25 (Skaanild, 2006; Hosseinpour and Wikvall, 2000). Furthermore, in men ketoconazole and sulphaphenazole do not significantly inhibit CYP2D6 (Cyrus et al, 2011)

# SPSS output multiple linear regression

## OH_TB [2] with enzymes

| **Model Summary^c,d^** | | | | | |
| --- | --- | --- | --- | --- | --- |
| Model | R | | R Square | Adjusted R Square | Std. Error of the Estimate |
|  | Concentration_ID = Concentration 2 (Selected) | Concentration_ID ~= Concentration 2 (Unselected) |  |  |  |
| 1 | ,652^a^ |  | ,425 | ,383 | ,54582 |
| 2 | ,755^b^ | ,437 | ,570 | ,504 | ,48972 |
| a. Predictors: (Constant), CYP2C49_s | | | | | |
| b. Predictors: (Constant), CYP2C49_s, CYP2A19_s | | | | | |
| c. Unless noted otherwise, statistics are based only on cases for which Concentration_ID = Concentration 2. | | | | | |
| d. Dependent Variable: OH_TB | | | | | |

| **Coefficients^a,b^** | | | | | | | | |
| --- | --- | --- | --- | --- | --- | --- | --- | --- |
| Model | | Unstandardized Coefficients | | Standardized Coefficients | t | Sig. | Collinearity Statistics | |
|  |  | B | Std. Error | Beta |  |  | Tolerance | VIF |
| 1 | (Constant) | 1,524 | ,136 |  | 11,169 | ,000 |  |  |
|  | CYP2C49_s | ,453 | ,141 | ,652 | 3,214 | ,006 | 1,000 | 1,000 |
| 2 | (Constant) | 1,524 | ,122 |  | 12,449 | ,000 |  |  |
|  | CYP2C49_s | ,508 | ,129 | ,731 | 3,935 | ,002 | ,958 | 1,044 |
|  | CYP2A19_s | -,271 | ,129 | -,389 | -2,096 | ,056 | ,958 | 1,044 |
| a. Dependent Variable: OH_TB | | | | | | | | |
| b. Selecting only cases for which Concentration_ID = Concentration 2 | | | | | | | | |

| **Excluded Variables^a^** | | | | | | | | |
| --- | --- | --- | --- | --- | --- | --- | --- | --- |
| Model | | Beta In | t | Sig. | Partial Correlation | Collinearity Statistics | | |
|  |  |  |  |  |  | Tolerance | VIF | Minimum Tolerance |
| 1 | CYP3A_s | -,038^b^ | -,178 | ,862 | -,049 | ,949 | 1,054 | ,949 |
|  | CYP3A22_s | ,120^b^ | ,540 | ,598 | ,148 | ,872 | 1,146 | ,872 |
|  | CYP1A2_s | ,012^b^ | ,056 | ,956 | ,016 | ,936 | 1,069 | ,936 |
|  | CYP2D25_s | -,065^b^ | -,306 | ,764 | -,085 | ,971 | 1,030 | ,971 |
|  | CYP3A46_s | ,100^b^ | ,475 | ,643 | ,131 | ,979 | 1,022 | ,979 |
|  | CYP2E1_s | ,083^b^ | ,365 | ,721 | ,101 | ,859 | 1,164 | ,859 |
|  | CYP2C33_s | ,305^b^ | 1,343 | ,202 | ,349 | ,755 | 1,324 | ,755 |
|  | CYP2A19_s | -,389^b^ | -2,096 | ,056 | -,502 | ,958 | 1,044 | ,958 |
| 2 | CYP3A_s | ,231^c^ | 1,058 | ,311 | ,292 | ,687 | 1,456 | ,687 |
|  | CYP3A22_s | ,254^c^ | 1,282 | ,224 | ,347 | ,805 | 1,243 | ,805 |
|  | CYP1A2_s | ,264^c^ | 1,255 | ,234 | ,341 | ,718 | 1,392 | ,718 |
|  | CYP2D25_s | ,149^c^ | ,697 | ,499 | ,197 | ,758 | 1,319 | ,748 |
|  | CYP3A46_s | ,292^c^ | 1,541 | ,149 | ,406 | ,833 | 1,200 | ,816 |
|  | CYP2E1_s | ,184^c^ | ,907 | ,382 | ,253 | ,816 | 1,226 | ,816 |
|  | CYP2C33_s | ,236^c^ | 1,122 | ,284 | ,308 | ,733 | 1,364 | ,704 |
| a. Dependent Variable: OH_TB | | | | | | | | |
| b. Predictors in the Model: (Constant), CYP2C49_s | | | | | | | | |
| c. Predictors in the Model: (Constant), CYP2C49_s, CYP2A19_s | | | | | | | | |

## OH_TB [2] with metabolites

| **Model Summary^b,c^** | | | | | |
| --- | --- | --- | --- | --- | --- |
| Model | R | | R Square | Adjusted R Square | Std. Error of the Estimate |
|  | Concentration_ID = Concentration 2 (Selected) | Concentration_ID ~= Concentration 2 (Unselected) |  |  |  |
| 1 | ,595^a^ | ,247 | ,354 | ,308 | ,57827 |
| a. Predictors: (Constant), DEX_s | | | | | |
| b. Unless noted otherwise, statistics are based only on cases for which Concentration_ID = Concentration 2. | | | | | |
| c. Dependent Variable: OH_TB | | | | | |

| **Coefficients^a,b^** | | | | | | | | |
| --- | --- | --- | --- | --- | --- | --- | --- | --- |
| Model | | Unstandardized Coefficients | | Standardized Coefficients | t | Sig. | Collinearity Statistics | |
|  |  | B | Std. Error | Beta |  |  | Tolerance | VIF |
| 1 | (Constant) | 1,524 | ,145 |  | 10,542 | ,000 |  |  |
|  | DEX_s | ,414 | ,149 | ,595 | 2,771 | ,015 | 1,000 | 1,000 |
| a. Dependent Variable: OH_TB | | | | | | | | |
| b. Selecting only cases for which Concentration_ID = Concentration 2 | | | | | | | | |

| **Excluded Variables^a^** | | | | | | | | |
| --- | --- | --- | --- | --- | --- | --- | --- | --- |
| Model | | Beta In | t | Sig. | Partial Correlation | Collinearity Statistics | | |
|  |  |  |  |  |  | Tolerance | VIF | Minimum Tolerance |
| 1 | OH_CZ_s | -,005^b^ | -,020 | ,984 | -,005 | ,949 | 1,053 | ,949 |
|  | OH_CM_s | -,231^b^ | -1,077 | ,301 | -,286 | ,990 | 1,010 | ,990 |
|  | PAR_s | -,021^b^ | -,093 | ,928 | -,026 | ,966 | 1,035 | ,966 |
|  | OH_MDZ_s | ,241^b^ | 1,012 | ,330 | ,270 | ,809 | 1,235 | ,809 |
| a. Dependent Variable: OH_TB | | | | | | | | |
| b. Predictors in the Model: (Constant), DEX_s | | | | | | | | |

## OH_TB [6] with Enzymes

| **Model Summary^d,e^** | | | | | |
| --- | --- | --- | --- | --- | --- |
| Model | R | | R Square | Adjusted R Square | Std. Error of the Estimate |
|  | Concentration_ID = Concentration 6 (Selected) | Concentration_ID ~= Concentration 6 (Unselected) |  |  |  |
| 1 | ,718^a^ |  | ,515 | ,481 | 12,54588 |
| 2 | ,853^b^ |  | ,727 | ,685 | 9,77312 |
| 3 | ,892^c^ | ,364 | ,797 | ,746 | 8,78091 |
| a. Predictors: (Constant), CYP2C49_s | | | | | |
| b. Predictors: (Constant), CYP2C49_s, CYP2A19_s | | | | | |
| c. Predictors: (Constant), CYP2C49_s, CYP2A19_s, CYP3A22_s | | | | | |
| d. Unless noted otherwise, statistics are based only on cases for which Concentration_ID = Concentration 6. | | | | | |
| e. Dependent Variable: OH_TB | | | | | |

| **Coefficientsa,b** | | | | | | | | | | | | | | |  |
| --- | --- | --- | --- | --- | --- | --- | --- | --- | --- | --- | --- | --- | --- | --- | --- |
| Model | | Unstandardized Coefficients | | | | Standardized Coefficients | | t | | Sig. | | Collinearity Statistics | | | |
|  |  | B | | Std. Error | | Beta | |  |  |  |  | Tolerance | | VIF | |
| 1 | (Constant) | 32,576 | | 3,136 | |  | | 10,386 | | ,000 | |  | |  | |
|  | CYP2C49_s | 12,502 | | 3,239 | | ,718 | | 3,859 | | ,002 | | 1,000 | | 1,000 | |
| 2 | (Constant) | 32,576 | | 2,443 | |  | | 13,333 | | ,000 | |  | |  | |
|  | CYP2C49_s | 14,174 | | 2,578 | | ,814 | | 5,498 | | ,000 | | ,958 | | 1,044 | |
|  | CYP2A19_s | -8,181 | | 2,578 | | -,470 | | -3,173 | | ,007 | | ,958 | | 1,044 | |
| 3 | (Constant) | 32,576 | | 2,195 | |  | | 14,840 | | ,000 | |  | |  | |
|  | CYP2C49_s | 12,624 | | 2,439 | | ,725 | | 5,175 | | ,000 | | ,864 | | 1,157 | |
|  | CYP2A19_s | -9,543 | | 2,412 | | -,548 | | -3,957 | | ,002 | | ,884 | | 1,132 | |
|  | CYP3A22_s | 5,120 | | 2,527 | | ,294 | | 2,026 | | ,066 | | ,805 | | 1,243 | |
| a. Dependent Variable: OH_TB | | | | | | | | | | | | | | | |
| b. Selecting only cases for which Concentration_ID = Concentration 6 | | | | | | | | | | | | | | | |
| **Excluded Variables^a^** | | | | | | | | | | | | | | |  |
| Model | | Beta In | t | | Sig. | | Partial Correlation | | Collinearity Statistics | | | | | |  |
|  |  |  |  |  |  |  |  |  | Tolerance | | VIF | | Minimum Tolerance | |  |
| 1 | CYP3A_s | -,057^b^ | -,290 | | ,777 | | -,080 | | ,949 | | 1,054 | | ,949 | |  |
|  | CYP3A22_s | ,134^b^ | ,659 | | ,522 | | ,180 | | ,872 | | 1,146 | | ,872 | |  |
|  | CYP1A2_s | -,010^b^ | -,052 | | ,960 | | -,014 | | ,936 | | 1,069 | | ,936 | |  |
|  | CYP2D25_s | -,076^b^ | -,391 | | ,702 | | -,108 | | ,971 | | 1,030 | | ,971 | |  |
|  | CYP3A46_s | ,015^b^ | ,079 | | ,939 | | ,022 | | ,979 | | 1,022 | | ,979 | |  |
|  | CYP2E1_s | ,096^b^ | ,464 | | ,650 | | ,128 | | ,859 | | 1,164 | | ,859 | |  |
|  | CYP2C33_s | ,339^b^ | 1,682 | | ,116 | | ,423 | | ,755 | | 1,324 | | ,755 | |  |
|  | CYP2A19_s | -,470^b^ | -3,173 | | ,007 | | -,661 | | ,958 | | 1,044 | | ,958 | |  |
| 2 | CYP3A_s | ,264^c^ | 1,594 | | ,137 | | ,418 | | ,687 | | 1,456 | | ,687 | |  |
|  | CYP3A22_s | ,294^c^ | 2,026 | | ,066 | | ,505 | | ,805 | | 1,243 | | ,805 | |  |
|  | CYP1A2_s | ,285^c^ | 1,808 | | ,096 | | ,463 | | ,718 | | 1,392 | | ,718 | |  |
|  | CYP2D25_s | ,182^c^ | 1,104 | | ,291 | | ,304 | | ,758 | | 1,319 | | ,748 | |  |
|  | CYP3A46_s | ,228^c^ | 1,508 | | ,158 | | ,399 | | ,833 | | 1,200 | | ,816 | |  |
|  | CYP2E1_s | ,218^c^ | 1,409 | | ,184 | | ,377 | | ,816 | | 1,226 | | ,816 | |  |
|  | CYP2C33_s | ,255^c^ | 1,593 | | ,137 | | ,418 | | ,733 | | 1,364 | | ,704 | |  |
| 3 | CYP3A_s | ,061^d^ | ,252 | | ,806 | | ,076 | | ,309 | | 3,231 | | ,309 | |  |
|  | CYP1A2_s | ,110^d^ | ,435 | | ,672 | | ,130 | | ,285 | | 3,505 | | ,285 | |  |
|  | CYP2D25_s | ,034^d^ | ,186 | | ,856 | | ,056 | | ,553 | | 1,808 | | ,553 | |  |
|  | CYP3A46_s | ,104^d^ | ,604 | | ,558 | | ,179 | | ,598 | | 1,671 | | ,578 | |  |
|  | CYP2E1_s | ,085^d^ | ,483 | | ,639 | | ,144 | | ,579 | | 1,728 | | ,571 | |  |
|  | CYP2C33_s | ,186^d^ | 1,201 | | ,255 | | ,341 | | ,681 | | 1,468 | | ,681 | |  |
| a. Dependent Variable: OH_TB | | | | | | | | | | | | | | |  |
| b. Predictors in the Model: (Constant), CYP2C49_s | | | | | | | | | | | | | | |  |
| c. Predictors in the Model: (Constant), CYP2C49_s, CYP2A19_s | | | | | | | | | | | | | | |  |
| d. Predictors in the Model: (Constant), CYP2C49_s, CYP2A19_s, CYP3A22_s | | | | | | | | | | | | | | |  |

## OH_TB [6] with metabolites

| **Model Summary^b,c^** | | | | | |
| --- | --- | --- | --- | --- | --- |
| Model | R | | R Square | Adjusted R Square | Std. Error of the Estimate |
|  | Concentration_ID = Concentration 6 (Selected) | Concentration_ID ~= Concentration 6 (Unselected) |  |  |  |
| 1 | ,838^a^ | ,327 | ,702 | ,680 | 9,84630 |
| a. Predictors: (Constant), OH_CZ_s | | | | | |
| b. Unless noted otherwise, statistics are based only on cases for which Concentration_ID = Concentration 6. | | | | | |
| c. Dependent Variable: OH_TB | | | | | |

| **Coefficients^a,b^** | | | | | | | | |
| --- | --- | --- | --- | --- | --- | --- | --- | --- |
| Model | | Unstandardized Coefficients | | Standardized Coefficients | t | Sig. | Collinearity Statistics | |
|  |  | B | Std. Error | Beta |  |  | Tolerance | VIF |
| 1 | (Constant) | 32,576 | 2,462 |  | 13,234 | ,000 |  |  |
|  | OH_CZ_s | 14,585 | 2,542 | ,838 | 5,737 | ,000 | 1,000 | 1,000 |
| a. Dependent Variable: OH_TB | | | | | | | | |
| b. Selecting only cases for which Concentration_ID = Concentration 6 | | | | | | | | |

| **Excluded Variables^a^** | | | | | | | | |
| --- | --- | --- | --- | --- | --- | --- | --- | --- |
| Model | | Beta In | t | Sig. | Partial Correlation | Collinearity Statistics | | |
|  |  |  |  |  |  | Tolerance | VIF | Minimum Tolerance |
| 1 | OH_CM_s | -,193^b^ | -1,365 | ,195 | -,354 | ,999 | 1,001 | ,999 |
|  | PAR_s | -,255^b^ | -1,361 | ,196 | -,353 | ,575 | 1,740 | ,575 |
|  | OH_MDZ_s | -,165^b^ | -,664 | ,518 | -,181 | ,361 | 2,769 | ,361 |
|  | DEX_s | -,068^b^ | -,388 | ,704 | -,107 | ,748 | 1,338 | ,748 |
| a. Dependent Variable: OH_TB | | | | | | | | |
| b. Predictors in the Model: (Constant), OH_CZ_s | | | | | | | | |

## OH_CZ [2] with enzymes

| **Model Summary^c,d^** | | | | | |
| --- | --- | --- | --- | --- | --- |
| Model | R | | R Square | Adjusted R Square | Std. Error of the Estimate |
|  | Concentration_ID = Concentration 2 (Selected) | Concentration_ID ~= Concentration 2 (Unselected) |  |  |  |
| 1 | ,497^a^ |  | ,247 | ,193 | 19,66660 |
| 2 | ,679^b^ | ,037 | ,461 | ,378 | 17,27232 |
| a. Predictors: (Constant), CYP3A46_s | | | | | |
| b. Predictors: (Constant), CYP3A46_s, CYP2C33_s | | | | | |
| c. Unless noted otherwise, statistics are based only on cases for which Concentration_ID = Concentration 2. | | | | | |
| d. Dependent Variable: OH_CZ | | | | | |

| **Coefficients^a,b^** | | | | | | | | |
| --- | --- | --- | --- | --- | --- | --- | --- | --- |
| Model | | Unstandardized Coefficients | | Standardized Coefficients | t | Sig. | Collinearity Statistics | |
|  |  | B | Std. Error | Beta |  |  | Tolerance | VIF |
| 1 | (Constant) | 80,093 | 4,917 |  | 16,290 | ,000 |  |  |
|  | CYP3A46_s | 10,880 | 5,078 | ,497 | 2,143 | ,050 | 1,000 | 1,000 |
| 2 | (Constant) | 80,093 | 4,318 |  | 18,548 | ,000 |  |  |
|  | CYP3A46_s | 17,331 | 5,289 | ,792 | 3,277 | ,006 | ,711 | 1,406 |
|  | CYP2C33_s | -12,002 | 5,289 | -,548 | -2,269 | ,041 | ,711 | 1,406 |
| a. Dependent Variable: OH_CZ | | | | | | | | |
| b. Selecting only cases for which Concentration_ID = Concentration 2 | | | | | | | | |

| **Excluded Variables^a^** | | | | | | | | |
| --- | --- | --- | --- | --- | --- | --- | --- | --- |
| Model | | Beta In | t | Sig. | Partial Correlation | Collinearity Statistics | | |
|  |  |  |  |  |  | Tolerance | VIF | Minimum Tolerance |
| 1 | CYP3A_s | -,181^b^ | -,459 | ,654 | -,126 | ,365 | 2,740 | ,365 |
|  | CYP2C49_s | -,092^b^ | -,381 | ,710 | -,105 | ,979 | 1,022 | ,979 |
|  | CYP3A22_s | -,177^b^ | -,603 | ,557 | -,165 | ,656 | 1,523 | ,656 |
|  | CYP1A2_s | -,390^b^ | -,899 | ,385 | -,242 | ,291 | 3,441 | ,291 |
|  | CYP2D25_s | -,441^b^ | -1,178 | ,260 | -,311 | ,374 | 2,677 | ,374 |
|  | CYP2E1_s | -,294^b^ | -1,022 | ,325 | -,273 | ,650 | 1,540 | ,650 |
|  | CYP2C33_s | -,548^b^ | -2,269 | ,041 | -,533 | ,711 | 1,406 | ,711 |
|  | CYP2A19_s | ,258^b^ | 1,019 | ,327 | ,272 | ,838 | 1,194 | ,838 |
| 2 | CYP3A_s | -,024^c^ | -,067 | ,947 | -,019 | ,349 | 2,863 | ,347 |
|  | CYP2C49_s | ,188^c^ | ,778 | ,451 | ,219 | ,735 | 1,360 | ,534 |
|  | CYP3A22_s | -,154^c^ | -,599 | ,561 | -,170 | ,655 | 1,526 | ,528 |
|  | CYP1A2_s | -,147^c^ | -,358 | ,726 | -,103 | ,265 | 3,777 | ,265 |
|  | CYP2D25_s | -,205^c^ | -,561 | ,585 | -,160 | ,329 | 3,038 | ,329 |
|  | CYP2E1_s | -,007^c^ | -,024 | ,981 | -,007 | ,485 | 2,060 | ,485 |
|  | CYP2A19_s | ,098^c^ | ,399 | ,697 | ,114 | ,741 | 1,350 | ,528 |
| a. Dependent Variable: OH_CZ | | | | | | | | |
| b. Predictors in the Model: (Constant), CYP3A46_s | | | | | | | | |
| c. Predictors in the Model: (Constant), CYP3A46_s, CYP2C33_s | | | | | | | | |

## OH_CZ [2] with metabolites

| **Model Summary^b,c^** | | | | | |
| --- | --- | --- | --- | --- | --- |
| Model | R | | R Square | Adjusted R Square | Std. Error of the Estimate |
|  | Concentration_ID = Concentration 2 (Selected) | Concentration_ID ~= Concentration 2 (Unselected) |  |  |  |
| 1 | ,959^a^ | ,386 | ,920 | ,915 | 6,39149 |
| a. Predictors: (Constant), PAR_s | | | | | |
| b. Unless noted otherwise, statistics are based only on cases for which Concentration_ID = Concentration 2. | | | | | |
| c. Dependent Variable: OH_CZ | | | | | |

| **Coefficients^a,b^** | | | | | | | | |
| --- | --- | --- | --- | --- | --- | --- | --- | --- |
| Model | | Unstandardized Coefficients | | Standardized Coefficients | t | Sig. | Collinearity Statistics | |
|  |  | B | Std. Error | Beta |  |  | Tolerance | VIF |
| 1 | (Constant) | 80,093 | 1,598 |  | 50,124 | ,000 |  |  |
|  | PAR_s | 21,005 | 1,650 | ,959 | 12,728 | ,000 | 1,000 | 1,000 |
| a. Dependent Variable: OH_CZ | | | | | | | | |
| b. Selecting only cases for which Concentration_ID = Concentration 2 | | | | | | | | |

| **Excluded Variables^a^** | | | | | | | | |
| --- | --- | --- | --- | --- | --- | --- | --- | --- |
| Model | | Beta In | t | Sig. | Partial Correlation | Collinearity Statistics | | |
|  |  |  |  |  |  | Tolerance | VIF | Minimum Tolerance |
| 1 | OH_TB_s | ,044^b^ | ,569 | ,579 | ,156 | ,992 | 1,008 | ,992 |
|  | OH_CM_s | -,049^b^ | -,292 | ,775 | -,081 | ,213 | 4,705 | ,213 |
|  | OH_MDZ_s | ,029^b^ | ,306 | ,765 | ,084 | ,694 | 1,440 | ,694 |
|  | DEX_s | ,050^b^ | ,640 | ,534 | ,175 | ,966 | 1,035 | ,966 |
| a. Dependent Variable: OH_CZ | | | | | | | | |
| b. Predictors in the Model: (Constant), PAR_s | | | | | | | | |

## OH_CZ [6] with enzymes

| **Model Summary^d,e^** | | | | | |
| --- | --- | --- | --- | --- | --- |
| Model | R | | R Square | Adjusted R Square | Std. Error of the Estimate |
|  | Concentration_ID = Concentration 6 (Selected) | Concentration_ID ~= Concentration 6 (Unselected) |  |  |  |
| 1 | ,596^a^ |  | ,355 | ,309 | 132,99877 |
| 2 | ,712^b^ |  | ,506 | ,430 | 120,76381 |
| 3 | ,804^c^ | ,185 | ,646 | ,558 | 106,38055 |
| a. Predictors: (Constant), CYP2E1_s | | | | | |
| b. Predictors: (Constant), CYP2E1_s, CYP2A19_s | | | | | |
| c. Predictors: (Constant), CYP2E1_s, CYP2A19_s, CYP2C49_s | | | | | |
| d. Unless noted otherwise, statistics are based only on cases for which Concentration_ID = Concentration 6. | | | | | |
| e. Dependent Variable: OH_CZ | | | | | |

| **Coefficients^a,b^** | | | | | | | | |
| --- | --- | --- | --- | --- | --- | --- | --- | --- |
| Model | | Unstandardized Coefficients | | Standardized Coefficients | t | Sig. | Collinearity Statistics | |
|  |  | B | Std. Error | Beta |  |  | Tolerance | VIF |
| 1 | (Constant) | 595,976 | 33,250 |  | 17,924 | ,000 |  |  |
|  | CYP2E1_s | 95,371 | 34,340 | ,596 | 2,777 | ,015 | 1,000 | 1,000 |
| 2 | (Constant) | 595,976 | 30,191 |  | 19,740 | ,000 |  |  |
|  | CYP2E1_s | 113,525 | 32,482 | ,709 | 3,495 | ,004 | ,922 | 1,085 |
|  | CYP2A19_s | -64,804 | 32,482 | -,405 | -1,995 | ,067 | ,922 | 1,085 |
| 3 | (Constant) | 595,976 | 26,595 |  | 22,409 | ,000 |  |  |
|  | CYP2E1_s | 91,073 | 30,410 | ,569 | 2,995 | ,011 | ,816 | 1,226 |
|  | CYP2A19_s | -71,805 | 28,793 | -,449 | -2,494 | ,028 | ,910 | 1,099 |
|  | CYP2C49_s | 65,016 | 29,822 | ,406 | 2,180 | ,050 | ,848 | 1,179 |
| a. Dependent Variable: OH_CZ | | | | | | | | |
| b. Selecting only cases for which Concentration_ID = Concentration 6 | | | | | | | | |

| **Excluded Variables^a^** | | | | | | | | |
| --- | --- | --- | --- | --- | --- | --- | --- | --- |
| Model | | Beta In | t | Sig. | Partial Correlation | Collinearity Statistics | | |
|  |  |  |  |  |  | Tolerance | VIF | Minimum Tolerance |
| 1 | CYP3A_s | -,347^b^ | -,978 | ,346 | -,262 | ,366 | 2,731 | ,366 |
|  | CYP2C49_s | ,354^b^ | 1,617 | ,130 | ,409 | ,859 | 1,164 | ,859 |
|  | CYP3A22_s | ,268^b^ | ,972 | ,349 | ,260 | ,609 | 1,641 | ,609 |
|  | CYP1A2_s | -,224^b^ | -,582 | ,570 | -,159 | ,326 | 3,069 | ,326 |
|  | CYP2D25_s | -,507^b^ | -1,658 | ,121 | -,418 | ,438 | 2,285 | ,438 |
|  | CYP3A46_s | ,001^b^ | ,004 | ,997 | ,001 | ,650 | 1,540 | ,650 |
|  | CYP2C33_s | ,253^b^ | ,880 | ,395 | ,237 | ,565 | 1,771 | ,565 |
|  | CYP2A19_s | -,405^b^ | -1,995 | ,067 | -,484 | ,922 | 1,085 | ,922 |
| 2 | CYP3A_s | ,016^c^ | ,041 | ,968 | ,012 | ,252 | 3,967 | ,252 |
|  | CYP2C49_s | ,406^c^ | 2,180 | ,050 | ,533 | ,848 | 1,179 | ,816 |
|  | CYP3A22_s | ,385^c^ | 1,599 | ,136 | ,419 | ,584 | 1,712 | ,584 |
|  | CYP1A2_s | ,164^c^ | ,401 | ,696 | ,115 | ,242 | 4,138 | ,242 |
|  | CYP2D25_s | -,310^c^ | -,944 | ,364 | -,263 | ,355 | 2,820 | ,355 |
|  | CYP3A46_s | ,164^c^ | ,632 | ,539 | ,179 | ,588 | 1,699 | ,588 |
|  | CYP2C33_s | ,098^c^ | ,347 | ,735 | ,100 | ,507 | 1,972 | ,468 |
| 3 | CYP3A_s | ,201^d^ | ,556 | ,589 | ,165 | ,238 | 4,196 | ,238 |
|  | CYP3A22_s | ,319^d^ | 1,470 | ,170 | ,405 | ,571 | 1,752 | ,571 |
|  | CYP1A2_s | ,318^d^ | ,887 | ,394 | ,258 | ,233 | 4,288 | ,233 |
|  | CYP2D25_s | -,159^d^ | -,516 | ,616 | -,154 | ,331 | 3,020 | ,331 |
|  | CYP3A46_s | ,240^d^ | 1,067 | ,309 | ,306 | ,576 | 1,735 | ,564 |
|  | CYP2C33_s | -,144^d^ | -,527 | ,609 | -,157 | ,420 | 2,380 | ,420 |
| a. Dependent Variable: OH_CZ | | | | | | | | |
| b. Predictors in the Model: (Constant), CYP2E1_s | | | | | | | | |
| c. Predictors in the Model: (Constant), CYP2E1_s, CYP2A19_s | | | | | | | | |
| d. Predictors in the Model: (Constant), CYP2E1_s, CYP2A19_s, CYP2C49_s | | | | | | | | |

## OH_CZ [6] with metabolites

| **Model Summary^c,d^** | | | | | |
| --- | --- | --- | --- | --- | --- |
| Model | R | | R Square | Adjusted R Square | Std. Error of the Estimate |
|  | Concentration_ID = Concentration 6 (Selected) | Concentration_ID ~= Concentration 6 (Unselected) |  |  |  |
| 1 | ,838^a^ |  | ,702 | ,680 | 90,48465 |
| 2 | ,913^b^ | ,266 | ,834 | ,808 | 70,03469 |
| a. Predictors: (Constant), OH_TB_s | | | | | |
| b. Predictors: (Constant), OH_TB_s, OH_MDZ_s | | | | | |
| c. Unless noted otherwise, statistics are based only on cases for which Concentration_ID = Concentration 6. | | | | | |
| d. Dependent Variable: OH_CZ | | | | | |

| **Coefficients^a,b^** | | | | | | | | |
| --- | --- | --- | --- | --- | --- | --- | --- | --- |
| Model | | Unstandardized Coefficients | | Standardized Coefficients | t | Sig. | Collinearity Statistics | |
|  |  | B | Std. Error | Beta |  |  | Tolerance | VIF |
| 1 | (Constant) | 595,976 | 22,621 |  | 26,346 | ,000 |  |  |
|  | OH_TB_s | 134,028 | 23,363 | ,838 | 5,737 | ,000 | 1,000 | 1,000 |
| 2 | (Constant) | 595,976 | 17,509 |  | 34,039 | ,000 |  |  |
|  | OH_TB_s | 89,207 | 22,819 | ,557 | 3,909 | ,002 | ,628 | 1,592 |
|  | OH_MDZ_s | 73,483 | 22,819 | ,459 | 3,220 | ,007 | ,628 | 1,592 |
| a. Dependent Variable: OH_CZ | | | | | | | | |
| b. Selecting only cases for which Concentration_ID = Concentration 6 | | | | | | | | |

| **Excluded Variables^a^** | | | | | | | | |
| --- | --- | --- | --- | --- | --- | --- | --- | --- |
| Model | | Beta In | t | Sig. | Partial Correlation | Collinearity Statistics | | |
|  |  |  |  |  |  | Tolerance | VIF | Minimum Tolerance |
| 1 | OH_CM_s | ,176^b^ | 1,207 | ,249 | ,318 | ,972 | 1,029 | ,972 |
|  | PAR_s | ,378^b^ | 2,952 | ,011 | ,633 | ,840 | 1,190 | ,840 |
|  | OH_MDZ_s | ,459^b^ | 3,220 | ,007 | ,666 | ,628 | 1,592 | ,628 |
|  | DEX_s | ,223^b^ | 1,476 | ,164 | ,379 | ,863 | 1,159 | ,863 |
| 2 | OH_CM_s | -,059^c^ | -,406 | ,692 | -,116 | ,642 | 1,558 | ,415 |
|  | PAR_s | ,178^c^ | ,924 | ,374 | ,258 | ,348 | 2,871 | ,260 |
|  | DEX_s | ,026^c^ | ,177 | ,863 | ,051 | ,630 | 1,588 | ,458 |
| a. Dependent Variable: OH_CZ | | | | | | | | |
| b. Predictors in the Model: (Constant), OH_TB_s | | | | | | | | |
| c. Predictors in the Model: (Constant), OH_TB_s, OH_MDZ_s | | | | | | | | |

## OH_CM [2] with enzymes

| **Model Summary^c,d^** | | | | | |
| --- | --- | --- | --- | --- | --- |
| Model | R | | R Square | Adjusted R Square | Std. Error of the Estimate |
|  | Concentration_ID = Concentration 2 (Selected) | Concentration_ID ~= Concentration 2 (Unselected) |  |  |  |
| 1 | ,465^a^ |  | ,217 | ,161 | 29,43424 |
| 2 | ,639^b^ | ,487 | ,408 | ,317 | 26,54095 |
| a. Predictors: (Constant), CYP2A19_s | | | | | |
| b. Predictors: (Constant), CYP2A19_s, CYP2C33_s | | | | | |
| c. Unless noted otherwise, statistics are based only on cases for which Concentration_ID = Concentration 2. | | | | | |
| d. Dependent Variable: OH_CM | | | | | |

| **Coefficients^a,b^** | | | | | | | | |
| --- | --- | --- | --- | --- | --- | --- | --- | --- |
| Model | | Unstandardized Coefficients | | Standardized Coefficients | t | Sig. | Collinearity Statistics | |
|  |  | B | Std. Error | Beta |  |  | Tolerance | VIF |
| 1 | (Constant) | 84,249 | 7,359 |  | 11,449 | ,000 |  |  |
|  | CYP2A19_s | 14,950 | 7,600 | ,465 | 1,967 | ,069 | 1,000 | 1,000 |
| 2 | (Constant) | 84,249 | 6,635 |  | 12,697 | ,000 |  |  |
|  | CYP2A19_s | 14,309 | 6,860 | ,445 | 2,086 | ,057 | ,998 | 1,002 |
|  | CYP2C33_s | -14,090 | 6,860 | -,439 | -2,054 | ,061 | ,998 | 1,002 |
| a. Dependent Variable: OH_CM | | | | | | | | |
| b. Selecting only cases for which Concentration_ID = Concentration 2 | | | | | | | | |

| **Excluded Variables^a^** | | | | | | | | |
| --- | --- | --- | --- | --- | --- | --- | --- | --- |
| Model | | Beta In | t | Sig. | Partial Correlation | Collinearity Statistics | | |
|  |  |  |  |  |  | Tolerance | VIF | Minimum Tolerance |
| 1 | CYP3A_s | -,338^b^ | -1,217 | ,245 | -,320 | ,701 | 1,427 | ,701 |
|  | CYP2C49_s | -,331^b^ | -1,420 | ,179 | -,366 | ,958 | 1,044 | ,958 |
|  | CYP3A22_s | -,415^b^ | -1,783 | ,098 | -,443 | ,893 | 1,120 | ,893 |
|  | CYP1A2_s | -,393^b^ | -1,490 | ,160 | -,382 | ,741 | 1,349 | ,741 |
|  | CYP2D25_s | -,308^b^ | -1,152 | ,270 | -,304 | ,763 | 1,310 | ,763 |
|  | CYP3A46_s | ,009^b^ | ,032 | ,975 | ,009 | ,838 | 1,194 | ,838 |
|  | CYP2E1_s | -,408^b^ | -1,778 | ,099 | -,442 | ,922 | 1,085 | ,922 |
|  | CYP2C33_s | -,439^b^ | -2,054 | ,061 | -,495 | ,998 | 1,002 | ,998 |
| 2 | CYP3A_s | ,021^c^ | ,059 | ,954 | ,017 | ,388 | 2,578 | ,388 |
|  | CYP2C49_s | -,137^c^ | -,524 | ,610 | -,149 | ,704 | 1,421 | ,704 |
|  | CYP3A22_s | -,280^c^ | -1,164 | ,267 | -,318 | ,765 | 1,307 | ,765 |
|  | CYP1A2_s | -,062^c^ | -,171 | ,867 | -,049 | ,367 | 2,725 | ,367 |
|  | CYP2D25_s | ,105^c^ | ,288 | ,778 | ,083 | ,372 | 2,690 | ,372 |
|  | CYP3A46_s | ,475^c^ | 1,742 | ,107 | ,449 | ,528 | 1,894 | ,528 |
|  | CYP2E1_s | -,173^c^ | -,538 | ,600 | -,154 | ,468 | 2,135 | ,468 |
| a. Dependent Variable: OH_CM | | | | | | | | |
| b. Predictors in the Model: (Constant), CYP2A19_s | | | | | | | | |
| c. Predictors in the Model: (Constant), CYP2A19_s, CYP2C33_s | | | | | | | | |

## OH_CM [2] with metabolites

| **Model Summary^c,d^** | | | | | |
| --- | --- | --- | --- | --- | --- |
| Model | R | | R Square | Adjusted R Square | Std. Error of the Estimate |
|  | Concentration_ID = Concentration 2 (Selected) | Concentration_ID ~= Concentration 2 (Unselected) |  |  |  |
| 1 | ,887^a^ |  | ,787 | ,772 | 15,33091 |
| 2 | ,961^b^ | ,574 | ,924 | ,913 | 9,49065 |
| a. Predictors: (Constant), PAR_s | | | | | |
| b. Predictors: (Constant), PAR_s, OH_TB_s | | | | | |
| c. Unless noted otherwise, statistics are based only on cases for which Concentration_ID = Concentration 2. | | | | | |
| d. Dependent Variable: OH_CM | | | | | |

| **Coefficients^a,b^** | | | | | | | | |
| --- | --- | --- | --- | --- | --- | --- | --- | --- |
| Model | | Unstandardized Coefficients | | Standardized Coefficients | t | Sig. | Collinearity Statistics | |
|  |  | B | Std. Error | Beta |  |  | Tolerance | VIF |
| 1 | (Constant) | 84,249 | 3,833 |  | 21,981 | ,000 |  |  |
|  | PAR_s | 28,509 | 3,958 | ,887 | 7,202 | ,000 | 1,000 | 1,000 |
| 2 | (Constant) | 84,249 | 2,373 |  | 35,508 | ,000 |  |  |
|  | PAR_s | 29,574 | 2,460 | ,921 | 12,020 | ,000 | ,992 | 1,008 |
|  | OH_TB_s | -11,935 | 2,460 | -,371 | -4,851 | ,000 | ,992 | 1,008 |
| a. Dependent Variable: OH_CM | | | | | | | | |
| b. Selecting only cases for which Concentration_ID = Concentration 2 | | | | | | | | |

| **Excluded Variables^a^** | | | | | | | | |
| --- | --- | --- | --- | --- | --- | --- | --- | --- |
| Model | | Beta In | t | Sig. | Partial Correlation | Collinearity Statistics | | |
|  |  |  |  |  |  | Tolerance | VIF | Minimum Tolerance |
| 1 | OH_TB_s | -,371^b^ | -4,851 | ,000 | -,803 | ,992 | 1,008 | ,992 |
|  | OH_CZ_s | -,132^b^ | -,292 | ,775 | -,081 | ,080 | 12,572 | ,080 |
|  | OH_MDZ_s | -,306^b^ | -2,390 | ,033 | -,552 | ,694 | 1,440 | ,694 |
|  | DEX_s | -,274^b^ | -2,598 | ,022 | -,585 | ,966 | 1,035 | ,966 |
| 2 | OH_CZ_s | ,074^c^ | ,261 | ,799 | ,075 | ,078 | 12,885 | ,078 |
|  | OH_MDZ_s | -,117^c^ | -1,123 | ,283 | -,308 | ,529 | 1,891 | ,529 |
|  | DEX_s | -,079^c^ | -,807 | ,435 | -,227 | ,627 | 1,595 | ,627 |
| a. Dependent Variable: OH_CM | | | | | | | | |
| b. Predictors in the Model: (Constant), PAR_s | | | | | | | | |
| c. Predictors in the Model: (Constant), PAR_s, OH_TB_s | | | | | | | | |

## OH_CM [6] with enzymes

| **Model Summary^b,c^** | | | | | |
| --- | --- | --- | --- | --- | --- |
| Model | R | | R Square | Adjusted R Square | Std. Error of the Estimate |
|  | Concentration_ID = Concentration 6 (Selected) | Concentration_ID ~= Concentration 6 (Unselected) |  |  |  |
| 1 | ,658^a^ | ,373 | ,434 | ,393 | 95,61393 |
| a. Predictors: (Constant), CYP2A19_s | | | | | |
| b. Unless noted otherwise, statistics are based only on cases for which Concentration_ID = Concentration 6. | | | | | |
| c. Dependent Variable: OH_CM | | | | | |

| **Coefficients^a,b^** | | | | | | | | |
| --- | --- | --- | --- | --- | --- | --- | --- | --- |
| Model | | Unstandardized Coefficients | | Standardized Coefficients | t | Sig. | Collinearity Statistics | |
|  |  | B | Std. Error | Beta |  |  | Tolerance | VIF |
| 1 | (Constant) | 302,082 | 23,903 |  | 12,638 | ,000 |  |  |
|  | CYP2A19_s | 80,819 | 24,687 | ,658 | 3,274 | ,006 | 1,000 | 1,000 |
| a. Dependent Variable: OH_CM | | | | | | | | |
| b. Selecting only cases for which Concentration_ID = Concentration 6 | | | | | | | | |

| **Excluded Variables^a^** | | | | | | | | |
| --- | --- | --- | --- | --- | --- | --- | --- | --- |
| Model | | Beta In | t | Sig. | Partial Correlation | Collinearity Statistics | | |
|  |  |  |  |  |  | Tolerance | VIF | Minimum Tolerance |
| 1 | CYP3A_s | -,207^b^ | -,852 | ,410 | -,230 | ,701 | 1,427 | ,701 |
|  | CYP2C49_s | ,012^b^ | ,058 | ,955 | ,016 | ,958 | 1,044 | ,958 |
|  | CYP3A22_s | -,194^b^ | -,904 | ,382 | -,243 | ,893 | 1,120 | ,893 |
|  | CYP1A2_s | -,210^b^ | -,892 | ,389 | -,240 | ,741 | 1,349 | ,741 |
|  | CYP2D25_s | -,230^b^ | -,997 | ,337 | -,267 | ,763 | 1,310 | ,763 |
|  | CYP3A46_s | ,003^b^ | ,013 | ,990 | ,003 | ,838 | 1,194 | ,838 |
|  | CYP2E1_s | -,197^b^ | -,936 | ,366 | -,251 | ,922 | 1,085 | ,922 |
|  | CYP2C33_s | -,229^b^ | -1,151 | ,270 | -,304 | ,998 | 1,002 | ,998 |
| a. Dependent Variable: OH_CM | | | | | | | | |
| b. Predictors in the Model: (Constant), CYP2A19_s | | | | | | | | |

## OH_CM [6] with metabolites

| **Model Summary^c,d^** | | | | | |
| --- | --- | --- | --- | --- | --- |
| Model | R | | R Square | Adjusted R Square | Std. Error of the Estimate |
|  | Concentration_ID = Concentration 6 (Selected) | Concentration_ID ~= Concentration 6 (Unselected) |  |  |  |
| 1 | ,663^a^ |  | ,440 | ,400 | 95,07761 |
| 2 | ,849^b^ | ,488 | ,721 | ,678 | 69,60655 |
| a. Predictors: (Constant), PAR_s | | | | | |
| b. Predictors: (Constant), PAR_s, OH_CZ_s | | | | | |
| c. Unless noted otherwise, statistics are based only on cases for which Concentration_ID = Concentration 6. | | | | | |
| d. Dependent Variable: OH_CM | | | | | |

| **Coefficients^a,b^** | | | | | | | | |
| --- | --- | --- | --- | --- | --- | --- | --- | --- |
| Model | | Unstandardized Coefficients | | Standardized Coefficients | t | Sig. | Collinearity Statistics | |
|  |  | B | Std. Error | Beta |  |  | Tolerance | VIF |
| 1 | (Constant) | 302,082 | 23,769 |  | 12,709 | ,000 |  |  |
|  | PAR_s | 81,407 | 24,549 | ,663 | 3,316 | ,005 | 1,000 | 1,000 |
| 2 | (Constant) | 302,082 | 17,402 |  | 17,359 | ,000 |  |  |
|  | PAR_s | 137,408 | 23,707 | 1,120 | 5,796 | ,000 | ,575 | 1,740 |
|  | OH_CZ_s | -85,873 | 23,707 | -,700 | -3,622 | ,003 | ,575 | 1,740 |
| a. Dependent Variable: OH_CM | | | | | | | | |
| b. Selecting only cases for which Concentration_ID = Concentration 6 | | | | | | | | |

| **Excluded Variables^a^** | | | | | | | | |
| --- | --- | --- | --- | --- | --- | --- | --- | --- |
| Model | | Beta In | t | Sig. | Partial Correlation | Collinearity Statistics | | |
|  |  |  |  |  |  | Tolerance | VIF | Minimum Tolerance |
| 1 | OH_TB_s | -,516^b^ | -2,936 | ,012 | -,631 | ,840 | 1,190 | ,840 |
|  | OH_CZ_s | -,700^b^ | -3,622 | ,003 | -,709 | ,575 | 1,740 | ,575 |
|  | OH_MDZ_s | -,493^b^ | -1,551 | ,145 | -,395 | ,361 | 2,773 | ,361 |
|  | DEX_s | -,347^b^ | -1,540 | ,148 | -,393 | ,718 | 1,393 | ,718 |
| 2 | OH_TB_s | -,113^c^ | -,381 | ,710 | -,109 | ,261 | 3,828 | ,179 |
|  | OH_MDZ_s | ,074^c^ | ,231 | ,821 | ,067 | ,226 | 4,418 | ,226 |
|  | DEX_s | -,207^c^ | -1,180 | ,261 | -,322 | ,676 | 1,480 | ,519 |
| a. Dependent Variable: OH_CM | | | | | | | | |
| b. Predictors in the Model: (Constant), PAR_s | | | | | | | | |
| c. Predictors in the Model: (Constant), PAR_s, OH_CZ_s | | | | | | | | |

## PAR [2] with enzymes

| **Model Summary^b,c^** | | | | | |
| --- | --- | --- | --- | --- | --- |
| Model | R | | R Square | Adjusted R Square | Std. Error of the Estimate |
|  | Concentration_ID = Concentration 2 (Selected) | Concentration_ID ~= Concentration 2 (Unselected) |  |  |  |
| 1 | ,515^a^ | ,213 | ,265 | ,213 | 114,03263 |
| a. Predictors: (Constant), CYP2A19_s | | | | | |
| b. Unless noted otherwise, statistics are based only on cases for which Concentration_ID = Concentration 2. | | | | | |
| c. Dependent Variable: PAR | | | | | |

| **Coefficients^a,b^** | | | | | | | | |
| --- | --- | --- | --- | --- | --- | --- | --- | --- |
| Model | | Unstandardized Coefficients | | Standardized Coefficients | t | Sig. | Collinearity Statistics | |
|  |  | B | Std. Error | Beta |  |  | Tolerance | VIF |
| 1 | (Constant) | 467,031 | 28,508 |  | 16,382 | ,000 |  |  |
|  | CYP2A19_s | 66,187 | 29,443 | ,515 | 2,248 | ,041 | 1,000 | 1,000 |
| a. Dependent Variable: PAR | | | | | | | | |
| b. Selecting only cases for which Concentration_ID = Concentration 2 | | | | | | | | |

| **Excluded Variables^a^** | | | | | | | | |
| --- | --- | --- | --- | --- | --- | --- | --- | --- |
| Model | | Beta In | t | Sig. | Partial Correlation | Collinearity Statistics | | |
|  |  |  |  |  |  | Tolerance | VIF | Minimum Tolerance |
| 1 | CYP3A_s | -,011^b^ | -,040 | ,968 | -,011 | ,701 | 1,427 | ,701 |
|  | CYP2C49_s | -,013^b^ | -,055 | ,957 | -,015 | ,958 | 1,044 | ,958 |
|  | CYP3A22_s | -,044^b^ | -,177 | ,862 | -,049 | ,893 | 1,120 | ,893 |
|  | CYP1A2_s | -,028^b^ | -,102 | ,921 | -,028 | ,741 | 1,349 | ,741 |
|  | CYP2D25_s | -,071^b^ | -,262 | ,797 | -,072 | ,763 | 1,310 | ,763 |
|  | CYP3A46_s | ,260^b^ | 1,043 | ,316 | ,278 | ,838 | 1,194 | ,838 |
|  | CYP2E1_s | -,073^b^ | -,296 | ,772 | -,082 | ,922 | 1,085 | ,922 |
|  | CYP2C33_s | -,148^b^ | -,631 | ,539 | -,172 | ,998 | 1,002 | ,998 |
| a. Dependent Variable: PAR | | | | | | | | |
| b. Predictors in the Model: (Constant), CYP2A19_s | | | | | | | | |

## PAR [2] with metabolites

| **Model Summary^d,e^** | | | | | |
| --- | --- | --- | --- | --- | --- |
| Model | R | | R Square | Adjusted R Square | Std. Error of the Estimate |
|  | Concentration_ID = Concentration 2 (Selected) | Concentration_ID ~= Concentration 2 (Unselected) |  |  |  |
| 1 | ,959^a^ |  | ,920 | ,915 | 37,51817 |
| 2 | ,971^b^ |  | ,943 | ,934 | 33,05568 |
| 3 | ,977^c^ | ,450 | ,955 | ,944 | 30,37997 |
| a. Predictors: (Constant), OH_CZ_s | | | | | |
| b. Predictors: (Constant), OH_CZ_s, OH_CM_s | | | | | |
| c. Predictors: (Constant), OH_CZ_s, OH_CM_s, OH_TB_s | | | | | |
| d. Unless noted otherwise, statistics are based only on cases for which Concentration_ID = Concentration 2. | | | | | |
| e. Dependent Variable: PAR | | | | | |

| **Coefficients^a,b^** | | | | | | | | |
| --- | --- | --- | --- | --- | --- | --- | --- | --- |
| Model | | Unstandardized Coefficients | | Standardized Coefficients | t | Sig. | Collinearity Statistics | |
|  |  | B | Std. Error | Beta |  |  | Tolerance | VIF |
| 1 | (Constant) | 467,031 | 9,380 |  | 49,792 | ,000 |  |  |
|  | OH_CZ_s | 123,303 | 9,687 | ,959 | 12,728 | ,000 | 1,000 | 1,000 |
| 2 | (Constant) | 467,031 | 8,264 |  | 56,514 | ,000 |  |  |
|  | OH_CZ_s | 93,549 | 15,769 | ,728 | 5,932 | ,000 | ,293 | 3,414 |
|  | OH_CM_s | 35,385 | 15,769 | ,275 | 2,244 | ,043 | ,293 | 3,414 |
| 3 | (Constant) | 467,031 | 7,595 |  | 61,492 | ,000 |  |  |
|  | OH_CZ_s | 65,915 | 20,862 | ,513 | 3,160 | ,008 | ,141 | 7,074 |
|  | OH_CM_s | 64,906 | 21,612 | ,505 | 3,003 | ,011 | ,132 | 7,591 |
|  | OH_TB_s | 21,722 | 11,796 | ,169 | 1,841 | ,090 | ,442 | 2,262 |
| a. Dependent Variable: PAR | | | | | | | | |
| b. Selecting only cases for which Concentration_ID = Concentration 2 | | | | | | | | |

| **Excluded Variables^a^** | | | | | | | | |
| --- | --- | --- | --- | --- | --- | --- | --- | --- |
| Model | | Beta In | t | Sig. | Partial Correlation | Collinearity Statistics | | |
|  |  |  |  |  |  | Tolerance | VIF | Minimum Tolerance |
| 1 | OH_TB_s | -,035^b^ | -,453 | ,658 | -,125 | ,983 | 1,017 | ,983 |
|  | OH_CM_s | ,275^b^ | 2,244 | ,043 | ,528 | ,293 | 3,414 | ,293 |
|  | OH_MDZ_s | ,036^b^ | ,384 | ,707 | ,106 | ,697 | 1,434 | ,697 |
|  | DEX_s | -,033^b^ | -,420 | ,681 | -,116 | ,949 | 1,053 | ,949 |
| 2 | OH_TB_s | ,169^c^ | 1,841 | ,090 | ,469 | ,442 | 2,262 | ,132 |
|  | OH_MDZ_s | ,130^c^ | 1,578 | ,140 | ,415 | ,581 | 1,721 | ,185 |
|  | DEX_s | ,073^c^ | ,887 | ,393 | ,248 | ,661 | 1,513 | ,196 |
| 3 | OH_MDZ_s | ,098^d^ | 1,208 | ,252 | ,342 | ,541 | 1,848 | ,125 |
|  | DEX_s | ,032^d^ | ,393 | ,702 | ,118 | ,594 | 1,684 | ,126 |
| a. Dependent Variable: PAR | | | | | | | | |
| b. Predictors in the Model: (Constant), OH_CZ_s | | | | | | | | |
| c. Predictors in the Model: (Constant), OH_CZ_s, OH_CM_s | | | | | | | | |
| d. Predictors in the Model: (Constant), OH_CZ_s, OH_CM_s, OH_TB_s | | | | | | | | |

## PAR [6] with enzymes

| **Model Summary^b,c^** | | | | | |
| --- | --- | --- | --- | --- | --- |
| Model | R | | R Square | Adjusted R Square | Std. Error of the Estimate |
|  | Concentration_ID = Concentration 6 (Selected) | Concentration_ID ~= Concentration 6 (Unselected) |  |  |  |
| 1 | ,512^a^ | ,265 | ,262 | ,210 | 266,95301 |
| a. Predictors: (Constant), CYP3A46_s | | | | | |
| b. Unless noted otherwise, statistics are based only on cases for which Concentration_ID = Concentration 6. | | | | | |
| c. Dependent Variable: PAR | | | | | |

| **Coefficients^a,b^** | | | | | | | | |
| --- | --- | --- | --- | --- | --- | --- | --- | --- |
| Model | | Unstandardized Coefficients | | Standardized Coefficients | t | Sig. | Collinearity Statistics | |
|  |  | B | Std. Error | Beta |  |  | Tolerance | VIF |
| 1 | (Constant) | 1281,345 | 66,738 |  | 19,200 | ,000 |  |  |
|  | CYP3A46_s | 153,831 | 68,927 | ,512 | 2,232 | ,042 | 1,000 | 1,000 |
| a. Dependent Variable: PAR | | | | | | | | |
| b. Selecting only cases for which Concentration_ID = Concentration 6 | | | | | | | | |

| **Excluded Variables^a^** | | | | | | | | |
| --- | --- | --- | --- | --- | --- | --- | --- | --- |
| Model | | Beta In | t | Sig. | Partial Correlation | Collinearity Statistics | | |
|  |  |  |  |  |  | Tolerance | VIF | Minimum Tolerance |
| 1 | CYP3A_s | -,103^b^ | -,261 | ,798 | -,072 | ,365 | 2,740 | ,365 |
|  | CYP2C49_s | ,238^b^ | 1,030 | ,322 | ,275 | ,979 | 1,022 | ,979 |
|  | CYP3A22_s | ,080^b^ | ,274 | ,788 | ,076 | ,656 | 1,523 | ,656 |
|  | CYP1A2_s | -,097^b^ | -,219 | ,830 | -,061 | ,291 | 3,441 | ,291 |
|  | CYP2D25_s | -,392^b^ | -1,048 | ,314 | -,279 | ,374 | 2,677 | ,374 |
|  | CYP2E1_s | ,074^b^ | ,253 | ,805 | ,070 | ,650 | 1,540 | ,650 |
|  | CYP2C33_s | -,056^b^ | -,199 | ,845 | -,055 | ,711 | 1,406 | ,711 |
|  | CYP2A19_s | ,035^b^ | ,136 | ,894 | ,038 | ,838 | 1,194 | ,838 |
| a. Dependent Variable: PAR | | | | | | | | |
| b. Predictors in the Model: (Constant), CYP3A46_s | | | | | | | | |

## PAR [6] with metabolites

| **Model Summary^e,f^** | | | | | |
| --- | --- | --- | --- | --- | --- |
| Model | R | | R Square | Adjusted R Square | Std. Error of the Estimate |
|  | Concentration_ID = Concentration 6 (Selected) | Concentration_ID ~= Concentration 6 (Unselected) |  |  |  |
| 1 | ,800^a^ |  | ,639 | ,614 | 186,66762 |
| 2 | ,897^b^ |  | ,805 | ,775 | 142,31482 |
| 3 | ,926^c^ |  | ,857 | ,822 | 126,82037 |
| 4 | ,916^d^ | ,439 | ,840 | ,815 | 129,16735 |
| a. Predictors: (Constant), OH_MDZ_s | | | | | |
| b. Predictors: (Constant), OH_MDZ_s, OH_CM_s | | | | | |
| c. Predictors: (Constant), OH_MDZ_s, OH_CM_s, OH_CZ_s | | | | | |
| d. Predictors: (Constant), OH_CM_s, OH_CZ_s | | | | | |
| e. Unless noted otherwise, statistics are based only on cases for which Concentration_ID = Concentration 6. | | | | | |
| f. Dependent Variable: PAR | | | | | |

| **Coefficients^a,b^** | | | | | | | | |
| --- | --- | --- | --- | --- | --- | --- | --- | --- |
| Model | | Unstandardized Coefficients | | Standardized Coefficients | t | Sig. | Collinearity Statistics | |
|  |  | B | Std. Error | Beta |  |  | Tolerance | VIF |
| 1 | (Constant) | 1281,345 | 46,667 |  | 27,457 | ,000 |  |  |
|  | OH_MDZ_s | 240,115 | 48,197 | ,800 | 4,982 | ,000 | 1,000 | 1,000 |
| 2 | (Constant) | 1281,345 | 35,579 |  | 36,014 | ,000 |  |  |
|  | OH_MDZ_s | 193,997 | 39,269 | ,646 | 4,940 | ,000 | ,876 | 1,142 |
|  | OH_CM_s | 130,750 | 39,269 | ,435 | 3,330 | ,005 | ,876 | 1,142 |
| 3 | (Constant) | 1281,345 | 31,705 |  | 40,414 | ,000 |  |  |
|  | OH_MDZ_s | 79,310 | 65,069 | ,264 | 1,219 | ,246 | ,253 | 3,949 |
|  | OH_CM_s | 167,326 | 39,124 | ,557 | 4,277 | ,001 | ,701 | 1,428 |
|  | OH_CZ_s | 127,350 | 60,915 | ,424 | 2,091 | ,059 | ,289 | 3,461 |
| 4 | (Constant) | 1281,345 | 32,292 |  | 39,680 | ,000 |  |  |
|  | OH_CM_s | 193,394 | 33,366 | ,644 | 5,796 | ,000 | ,999 | 1,001 |
|  | OH_CZ_s | 189,946 | 33,366 | ,633 | 5,693 | ,000 | ,999 | 1,001 |
| a. Dependent Variable: PAR | | | | | | | | |
| b. Selecting only cases for which Concentration_ID = Concentration 6 | | | | | | | | |

| **Excluded Variables^a^** | | | | | | | | |
| --- | --- | --- | --- | --- | --- | --- | --- | --- |
| Model | | Beta In | t | Sig. | Partial Correlation | Collinearity Statistics | | |
|  |  |  |  |  |  | Tolerance | VIF | Minimum Tolerance |
| 1 | OH_TB_s | -,140^b^ | -,677 | ,510 | -,184 | ,628 | 1,592 | ,628 |
|  | OH_CZ_s | ,036^b^ | ,130 | ,898 | ,036 | ,361 | 2,769 | ,361 |
|  | OH_CM_s | ,435^b^ | 3,330 | ,005 | ,678 | ,876 | 1,142 | ,876 |
|  | DEX_s | ,071^b^ | ,338 | ,741 | ,093 | ,630 | 1,588 | ,630 |
| 2 | OH_TB_s | ,171^c^ | ,947 | ,362 | ,264 | ,460 | 2,172 | ,415 |
|  | OH_CZ_s | ,424^c^ | 2,091 | ,059 | ,517 | ,289 | 3,461 | ,253 |
|  | DEX_s | ,151^c^ | ,966 | ,353 | ,269 | ,615 | 1,625 | ,545 |
| 3 | OH_TB_s | -,088^d^ | -,396 | ,700 | -,119 | ,261 | 3,832 | ,164 |
|  | DEX_s | ,162^d^ | 1,188 | ,260 | ,337 | ,615 | 1,627 | ,210 |
| 4 | OH_TB_s | -,084^e^ | -,371 | ,717 | -,107 | ,261 | 3,831 | ,261 |
|  | DEX_s | ,199^e^ | 1,633 | ,128 | ,426 | ,740 | 1,352 | ,740 |
|  | OH_MDZ_s | ,264^e^ | 1,219 | ,246 | ,332 | ,253 | 3,949 | ,253 |
| a. Dependent Variable: PAR | | | | | | | | |
| b. Predictors in the Model: (Constant), OH_MDZ_s | | | | | | | | |
| c. Predictors in the Model: (Constant), OH_MDZ_s, OH_CM_s | | | | | | | | |
| d. Predictors in the Model: (Constant), OH_MDZ_s, OH_CM_s, OH_CZ_s | | | | | | | | |
| e. Predictors in the Model: (Constant), OH_CM_s, OH_CZ_s | | | | | | | | |

## OH_MDZ [2] with enzymes

| **Model Summary^c,d^** | | | | | |
| --- | --- | --- | --- | --- | --- |
| Model | R | | R Square | Adjusted R Square | Std. Error of the Estimate |
|  | Concentration_ID = Concentration 2 (Selected) | Concentration_ID ~= Concentration 2 (Unselected) |  |  |  |
| 1 | ,616^a^ |  | ,380 | ,336 | 34,69639 |
| 2 | ,714^b^ | ,226 | ,509 | ,434 | 32,03478 |
| a. Predictors: (Constant), CYP3A22_s | | | | | |
| b. Predictors: (Constant), CYP3A22_s, CYP2E1_s | | | | | |
| c. Unless noted otherwise, statistics are based only on cases for which Concentration_ID = Concentration 2. | | | | | |
| d. Dependent Variable: OH_MDZ | | | | | |

| **Coefficients^a,b^** | | | | | | | | |
| --- | --- | --- | --- | --- | --- | --- | --- | --- |
| Model | | Unstandardized Coefficients | | Standardized Coefficients | t | Sig. | Collinearity Statistics | |
|  |  | B | Std. Error | Beta |  |  | Tolerance | VIF |
| 1 | (Constant) | 134,521 | 8,674 |  | 15,508 | ,000 |  |  |
|  | CYP3A22_s | 26,246 | 8,959 | ,616 | 2,930 | ,011 | 1,000 | 1,000 |
| 2 | (Constant) | 134,521 | 8,009 |  | 16,797 | ,000 |  |  |
|  | CYP3A22_s | 38,497 | 10,595 | ,904 | 3,633 | ,003 | ,609 | 1,641 |
|  | CYP2E1_s | -19,603 | 10,595 | -,460 | -1,850 | ,087 | ,609 | 1,641 |
| a. Dependent Variable: OH_MDZ | | | | | | | | |
| b. Selecting only cases for which Concentration_ID = Concentration 2 | | | | | | | | |

| **Excluded Variables^a^** | | | | | | | | |
| --- | --- | --- | --- | --- | --- | --- | --- | --- |
| Model | | Beta In | t | Sig. | Partial Correlation | Collinearity Statistics | | |
|  |  |  |  |  |  | Tolerance | VIF | Minimum Tolerance |
| 1 | CYP3A_s | -,499^b^ | -1,612 | ,131 | -,408 | ,415 | 2,412 | ,415 |
|  | CYP2C49_s | ,104^b^ | ,446 | ,663 | ,123 | ,872 | 1,146 | ,872 |
|  | CYP1A2_s | -,572^b^ | -1,737 | ,106 | -,434 | ,357 | 2,805 | ,357 |
|  | CYP2D25_s | -,365^b^ | -1,461 | ,168 | -,376 | ,655 | 1,526 | ,655 |
|  | CYP3A46_s | ,106^b^ | ,396 | ,699 | ,109 | ,656 | 1,523 | ,656 |
|  | CYP2E1_s | -,460^b^ | -1,850 | ,087 | -,457 | ,609 | 1,641 | ,609 |
|  | CYP2C33_s | -,277^b^ | -1,264 | ,228 | -,331 | ,883 | 1,132 | ,883 |
|  | CYP2A19_s | -,041^b^ | -,176 | ,863 | -,049 | ,893 | 1,120 | ,893 |
| 2 | CYP3A_s | -,243^c^ | -,609 | ,554 | -,173 | ,249 | 4,021 | ,249 |
|  | CYP2C49_s | ,192^c^ | ,897 | ,387 | ,251 | ,834 | 1,198 | ,583 |
|  | CYP1A2_s | -,301^c^ | -,658 | ,523 | -,187 | ,189 | 5,298 | ,189 |
|  | CYP2D25_s | -,152^c^ | -,488 | ,634 | -,140 | ,415 | 2,412 | ,385 |
|  | CYP3A46_s | ,303^c^ | 1,200 | ,253 | ,327 | ,573 | 1,746 | ,532 |
|  | CYP2C33_s | -,071^c^ | -,262 | ,798 | -,075 | ,557 | 1,797 | ,384 |
|  | CYP2A19_s | -,002^c^ | -,008 | ,993 | -,002 | ,883 | 1,132 | ,584 |
| a. Dependent Variable: OH_MDZ | | | | | | | | |
| b. Predictors in the Model: (Constant), CYP3A22_s | | | | | | | | |
| c. Predictors in the Model: (Constant), CYP3A22_s, CYP2E1_s | | | | | | | | |

## OH_MDZ [2] with metabolites

| **Model Summary^c,d^** | | | | | |
| --- | --- | --- | --- | --- | --- |
| Model | R | | R Square | Adjusted R Square | Std. Error of the Estimate |
|  | Concentration_ID = Concentration 2 (Selected) | Concentration_ID ~= Concentration 2 (Unselected) |  |  |  |
| 1 | ,553^a^ |  | ,306 | ,256 | 36,71841 |
| 2 | ,719^b^ | ,384 | ,518 | ,443 | 31,76170 |
| a. Predictors: (Constant), PAR_s | | | | | |
| b. Predictors: (Constant), PAR_s, OH_CM_s | | | | | |
| c. Unless noted otherwise, statistics are based only on cases for which Concentration_ID = Concentration 2. | | | | | |
| d. Dependent Variable: OH_MDZ | | | | | |

| **Coefficients^a,b^** | | | | | | | | |
| --- | --- | --- | --- | --- | --- | --- | --- | --- |
| Model | | Unstandardized Coefficients | | Standardized Coefficients | t | Sig. | Collinearity Statistics | |
|  |  | B | Std. Error | Beta |  |  | Tolerance | VIF |
| 1 | (Constant) | 134,521 | 9,180 |  | 14,654 | ,000 |  |  |
|  | PAR_s | 23,539 | 9,481 | ,553 | 2,483 | ,026 | 1,000 | 1,000 |
| 2 | (Constant) | 134,521 | 7,940 |  | 16,941 | ,000 |  |  |
|  | PAR_s | 61,260 | 17,788 | 1,439 | 3,444 | ,004 | ,213 | 4,705 |
|  | OH_CM_s | -42,509 | 17,788 | -,998 | -2,390 | ,033 | ,213 | 4,705 |
| a. Dependent Variable: OH_MDZ | | | | | | | | |
| b. Selecting only cases for which Concentration_ID = Concentration 2 | | | | | | | | |

| **Excluded Variables^a^** | | | | | | | | |
| --- | --- | --- | --- | --- | --- | --- | --- | --- |
| Model | | Beta In | t | Sig. | Partial Correlation | Collinearity Statistics | | |
|  |  |  |  |  |  | Tolerance | VIF | Minimum Tolerance |
| 1 | OH_TB_s | ,409^b^ | 2,018 | ,065 | ,488 | ,992 | 1,008 | ,992 |
|  | OH_CZ_s | ,249^b^ | ,306 | ,765 | ,084 | ,080 | 12,572 | ,080 |
|  | OH_CM_s | -,998^b^ | -2,390 | ,033 | -,552 | ,213 | 4,705 | ,213 |
|  | DEX_s | ,346^b^ | 1,614 | ,131 | ,409 | ,966 | 1,035 | ,966 |
| 2 | OH_TB_s | ,106^c^ | ,314 | ,759 | ,090 | ,353 | 2,833 | ,076 |
|  | OH_CZ_s | ,118^c^ | ,166 | ,871 | ,048 | ,079 | 12,655 | ,057 |
|  | DEX_s | ,110^c^ | ,442 | ,666 | ,127 | ,636 | 1,572 | ,137 |
| a. Dependent Variable: OH_MDZ | | | | | | | | |
| b. Predictors in the Model: (Constant), PAR_s | | | | | | | | |
| c. Predictors in the Model: (Constant), PAR_s, OH_CM_s | | | | | | | | |

## OH_MDZ [6] with enzymes

| **Model Summary^c,d^** | | | | | |
| --- | --- | --- | --- | --- | --- |
| Model | R | | R Square | Adjusted R Square | Std. Error of the Estimate |
|  | Concentration_ID = Concentration 6 (Selected) | Concentration_ID ~= Concentration 6 (Unselected) |  |  |  |
| 1 | ,676^a^ |  | ,458 | ,419 | 343,33116 |
| 2 | ,767^b^ | ,196 | ,589 | ,525 | 310,32054 |
| a. Predictors: (Constant), CYP3A46_s | | | | | |
| b. Predictors: (Constant), CYP3A46_s, CYP2C49_s | | | | | |
| c. Unless noted otherwise, statistics are based only on cases for which Concentration_ID = Concentration 6. | | | | | |
| d. Dependent Variable: OH_MDZ | | | | | |

| **ANOVA^a,b^** | | | | | | |
| --- | --- | --- | --- | --- | --- | --- |
| Model | | Sum of Squares | df | Mean Square | F | Sig. |
| 1 | Regression | 1392346,426 | 1 | 1392346,426 | 11,812 | ,004^c^ |
|  | Residual | 1650267,999 | 14 | 117876,286 |  |  |
|  | Total | 3042614,425 | 15 |  |  |  |
| 2 | Regression | 1790729,510 | 2 | 895364,755 | 9,298 | ,003^d^ |
|  | Residual | 1251884,915 | 13 | 96298,840 |  |  |
|  | Total | 3042614,425 | 15 |  |  |  |
| a. Dependent Variable: OH_MDZ | | | | | | |
| b. Selecting only cases for which Concentration_ID = Concentration 6 | | | | | | |
| c. Predictors: (Constant), CYP3A46_s | | | | | | |
| d. Predictors: (Constant), CYP3A46_s, CYP2C49_s | | | | | | |

| **Coefficients^a,b^** | | | | | | | | |
| --- | --- | --- | --- | --- | --- | --- | --- | --- |
| Model | | Unstandardized Coefficients | | Standardized Coefficients | t | Sig. | Collinearity Statistics | |
|  |  | B | Std. Error | Beta |  |  | Tolerance | VIF |
| 1 | (Constant) | 1478,221 | 85,833 |  | 17,222 | ,000 |  |  |
|  | CYP3A46_s | 304,669 | 88,648 | ,676 | 3,437 | ,004 | 1,000 | 1,000 |
| 2 | (Constant) | 1478,221 | 77,580 |  | 19,054 | ,000 |  |  |
|  | CYP3A46_s | 280,664 | 80,989 | ,623 | 3,465 | ,004 | ,979 | 1,022 |
|  | CYP2C49_s | 164,727 | 80,989 | ,366 | 2,034 | ,063 | ,979 | 1,022 |
| a. Dependent Variable: OH_MDZ | | | | | | | | |
| b. Selecting only cases for which Concentration_ID = Concentration 6 | | | | | | | | |

| **Excluded Variables^a^** | | | | | | | | |
| --- | --- | --- | --- | --- | --- | --- | --- | --- |
| Model | | Beta In | t | Sig. | Partial Correlation | Collinearity Statistics | | |
|  |  |  |  |  |  | Tolerance | VIF | Minimum Tolerance |
| 1 | CYP3A_s | -,121^b^ | -,358 | ,726 | -,099 | ,365 | 2,740 | ,365 |
|  | CYP2C49_s | ,366^b^ | 2,034 | ,063 | ,491 | ,979 | 1,022 | ,979 |
|  | CYP3A22_s | ,382^b^ | 1,669 | ,119 | ,420 | ,656 | 1,523 | ,656 |
|  | CYP1A2_s | -,032^b^ | -,085 | ,933 | -,024 | ,291 | 3,441 | ,291 |
|  | CYP2D25_s | -,325^b^ | -1,009 | ,332 | -,269 | ,374 | 2,677 | ,374 |
|  | CYP2E1_s | ,114^b^ | ,455 | ,657 | ,125 | ,650 | 1,540 | ,650 |
|  | CYP2C33_s | ,077^b^ | ,317 | ,756 | ,088 | ,711 | 1,406 | ,711 |
|  | CYP2A19_s | -,194^b^ | -,893 | ,388 | -,241 | ,838 | 1,194 | ,838 |
| 2 | CYP3A_s | -,239^c^ | -,785 | ,448 | -,221 | ,353 | 2,836 | ,353 |
|  | CYP3A22_s | ,260^c^ | 1,127 | ,282 | ,309 | ,581 | 1,721 | ,581 |
|  | CYP1A2_s | -,209^c^ | -,600 | ,560 | -,171 | ,273 | 3,662 | ,273 |
|  | CYP2D25_s | -,381^c^ | -1,345 | ,203 | -,362 | ,370 | 2,699 | ,370 |
|  | CYP2E1_s | -,056^c^ | -,227 | ,824 | -,065 | ,564 | 1,773 | ,564 |
|  | CYP2C33_s | -,183^c^ | -,739 | ,474 | -,209 | ,534 | 1,872 | ,534 |
|  | CYP2A19_s | -,264^c^ | -1,387 | ,191 | -,372 | ,816 | 1,226 | ,816 |
| a. Dependent Variable: OH_MDZ | | | | | | | | |
| b. Predictors in the Model: (Constant), CYP3A46_s | | | | | | | | |
| c. Predictors in the Model: (Constant), CYP3A46_s, CYP2C49_s | | | | | | | | |

## OH_MDZ [6] with metabolites

| **Model Summary^c,d^** | | | | | |
| --- | --- | --- | --- | --- | --- |
| Model | R | | R Square | Adjusted R Square | Std. Error of the Estimate |
|  | Concentration_ID = Concentration 6 (Selected) | Concentration_ID ~= Concentration 6 (Unselected) |  |  |  |
| 1 | ,800^a^ |  | ,639 | ,614 | 279,96185 |
| 2 | ,880^b^ | ,304 | ,774 | ,739 | 230,16629 |
| a. Predictors: (Constant), PAR_s | | | | | |
| b. Predictors: (Constant), PAR_s, OH_CZ_s | | | | | |
| c. Unless noted otherwise, statistics are based only on cases for which Concentration_ID = Concentration 6. | | | | | |
| d. Dependent Variable: OH_MDZ | | | | | |

| **Coefficients^a,b^** | | | | | | | | |
| --- | --- | --- | --- | --- | --- | --- | --- | --- |
| Model | | Unstandardized Coefficients | | Standardized Coefficients | t | Sig. | Collinearity Statistics | |
|  |  | B | Std. Error | Beta |  |  | Tolerance | VIF |
| 1 | (Constant) | 1478,221 | 69,990 |  | 21,120 | ,000 |  |  |
|  | PAR_s | 360,122 | 72,286 | ,800 | 4,982 | ,000 | 1,000 | 1,000 |
| 2 | (Constant) | 1478,221 | 57,542 |  | 25,690 | ,000 |  |  |
|  | PAR_s | 218,144 | 78,391 | ,484 | 2,783 | ,016 | ,575 | 1,740 |
|  | OH_CZ_s | 217,711 | 78,391 | ,483 | 2,777 | ,016 | ,575 | 1,740 |
| a. Dependent Variable: OH_MDZ | | | | | | | | |
| b. Selecting only cases for which Concentration_ID = Concentration 6 | | | | | | | | |

| **Excluded Variables^a^** | | | | | | | | |
| --- | --- | --- | --- | --- | --- | --- | --- | --- |
| Model | | Beta In | t | Sig. | Partial Correlation | Collinearity Statistics | | |
|  |  |  |  |  |  | Tolerance | VIF | Minimum Tolerance |
| 1 | OH_TB_s | ,345^b^ | 2,237 | ,043 | ,527 | ,840 | 1,190 | ,840 |
|  | OH_CZ_s | ,483^b^ | 2,777 | ,016 | ,610 | ,575 | 1,740 | ,575 |
|  | OH_CM_s | -,317^b^ | -1,551 | ,145 | -,395 | ,560 | 1,785 | ,560 |
|  | DEX_s | ,256^b^ | 1,398 | ,186 | ,361 | ,718 | 1,393 | ,718 |
| 2 | OH_TB_s | ,044^c^ | ,162 | ,874 | ,047 | ,261 | 3,828 | ,179 |
|  | OH_CM_s | ,060^c^ | ,231 | ,821 | ,067 | ,279 | 3,588 | ,160 |
|  | DEX_s | ,161^c^ | 1,001 | ,337 | ,278 | ,676 | 1,480 | ,519 |
| a. Dependent Variable: OH_MDZ | | | | | | | | |
| b. Predictors in the Model: (Constant), PAR_s | | | | | | | | |
| c. Predictors in the Model: (Constant), PAR_s, OH_CZ_s | | | | | | | | |

## DEX [2] with enzymes

| **Model Summary^c,d^** | | | | | |
| --- | --- | --- | --- | --- | --- |
| Model | R | | R Square | Adjusted R Square | Std. Error of the Estimate |
|  | Concentration_ID = Concentration 2 (Selected) | Concentration_ID ~= Concentration 2 (Unselected) |  |  |  |
| 1 | ,654^a^ |  | ,428 | ,387 | 70,41417 |
| 2 | ,746^b^ | ,365 | ,556 | ,488 | 64,37650 |
| a. Predictors: (Constant), CYP2C33_s | | | | | |
| b. Predictors: (Constant), CYP2C33_s, CYP3A46_s | | | | | |
| c. Unless noted otherwise, statistics are based only on cases for which Concentration_ID = Concentration 2. | | | | | |
| d. Dependent Variable: DEX | | | | | |

| **Coefficients^a,b^** | | | | | | | | |
| --- | --- | --- | --- | --- | --- | --- | --- | --- |
| Model | | Unstandardized Coefficients | | Standardized Coefficients | t | Sig. | Collinearity Statistics | |
|  |  | B | Std. Error | Beta |  |  | Tolerance | VIF |
| 1 | (Constant) | 188,676 | 17,604 |  | 10,718 | ,000 |  |  |
|  | CYP2C33_s | 58,821 | 18,181 | ,654 | 3,235 | ,006 | 1,000 | 1,000 |
| 2 | (Constant) | 188,676 | 16,094 |  | 11,723 | ,000 |  |  |
|  | CYP2C33_s | 38,307 | 19,711 | ,426 | 1,943 | ,074 | ,711 | 1,406 |
|  | CYP3A46_s | 38,167 | 19,711 | ,424 | 1,936 | ,075 | ,711 | 1,406 |
| a. Dependent Variable: DEX | | | | | | | | |
| b. Selecting only cases for which Concentration_ID = Concentration 2 | | | | | | | | |

| **Excluded Variables^a^** | | | | | | | | |
| --- | --- | --- | --- | --- | --- | --- | --- | --- |
| Model | | Beta In | t | Sig. | Partial Correlation | Collinearity Statistics | | |
|  |  |  |  |  |  | Tolerance | VIF | Minimum Tolerance |
| 1 | CYP3A_s | ,238^b^ | ,995 | ,338 | ,266 | ,715 | 1,398 | ,715 |
|  | CYP2C49_s | ,120^b^ | ,502 | ,624 | ,138 | ,755 | 1,324 | ,755 |
|  | CYP3A22_s | ,207^b^ | ,959 | ,355 | ,257 | ,883 | 1,132 | ,883 |
|  | CYP1A2_s | ,149^b^ | ,581 | ,571 | ,159 | ,654 | 1,529 | ,654 |
|  | CYP2D25_s | ,241^b^ | ,949 | ,360 | ,254 | ,636 | 1,571 | ,636 |
|  | CYP3A46_s | ,424^b^ | 1,936 | ,075 | ,473 | ,711 | 1,406 | ,711 |
|  | CYP2E1_s | ,250^b^ | ,926 | ,371 | ,249 | ,565 | 1,771 | ,565 |
|  | CYP2A19_s | -,054^b^ | -,260 | ,799 | -,072 | ,998 | 1,002 | ,998 |
| 2 | CYP3A_s | -,133^c^ | -,410 | ,689 | -,118 | ,349 | 2,863 | ,347 |
|  | CYP2C49_s | ,193^c^ | ,886 | ,393 | ,248 | ,735 | 1,360 | ,534 |
|  | CYP3A22_s | ,018^c^ | ,077 | ,940 | ,022 | ,655 | 1,526 | ,528 |
|  | CYP1A2_s | -,476^c^ | -1,369 | ,196 | -,368 | ,265 | 3,777 | ,265 |
|  | CYP2D25_s | -,136^c^ | -,409 | ,690 | -,117 | ,329 | 3,038 | ,329 |
|  | CYP2E1_s | ,084^c^ | ,305 | ,766 | ,088 | ,485 | 2,060 | ,485 |
|  | CYP2A19_s | -,318^c^ | -1,561 | ,144 | -,411 | ,741 | 1,350 | ,528 |
| a. Dependent Variable: DEX | | | | | | | | |
| b. Predictors in the Model: (Constant), CYP2C33_s | | | | | | | | |
| c. Predictors in the Model: (Constant), CYP2C33_s, CYP3A46_s | | | | | | | | |

## DEX [2] with metabolites

| **Model Summary^b,c^** | | | | | |
| --- | --- | --- | --- | --- | --- |
| Model | R | | R Square | Adjusted R Square | Std. Error of the Estimate |
|  | Concentration_ID = Concentration 2 (Selected) | Concentration_ID ~= Concentration 2 (Unselected) |  |  |  |
| 1 | ,596^a^ | ,217 | ,356 | ,310 | 74,72144 |
| a. Predictors: (Constant), OH_TB_s | | | | | |
| b. Unless noted otherwise, statistics are based only on cases for which Concentration_ID = Concentration 2. | | | | | |
| c. Dependent Variable: DEX | | | | | |

| **Coefficients^a,b^** | | | | | | | | |
| --- | --- | --- | --- | --- | --- | --- | --- | --- |
| Model | | Unstandardized Coefficients | | Standardized Coefficients | t | Sig. | Collinearity Statistics | |
|  |  | B | Std. Error | Beta |  |  | Tolerance | VIF |
| 1 | (Constant) | 188,676 | 18,680 |  | 10,100 | ,000 |  |  |
|  | OH_TB_s | 53,633 | 19,293 | ,596 | 2,780 | ,015 | 1,000 | 1,000 |
| a. Dependent Variable: DEX | | | | | | | | |
| b. Selecting only cases for which Concentration_ID = Concentration 2 | | | | | | | | |

| **Excluded Variables^a^** | | | | | | | | |
| --- | --- | --- | --- | --- | --- | --- | --- | --- |
| Model | | Beta In | t | Sig. | Partial Correlation | Collinearity Statistics | | |
|  |  |  |  |  |  | Tolerance | VIF | Minimum Tolerance |
| 1 | OH_CZ_s | ,151^b^ | ,683 | ,507 | ,186 | ,983 | 1,017 | ,983 |
|  | OH_CM_s | ,078^b^ | ,336 | ,742 | ,093 | ,916 | 1,091 | ,916 |
|  | PAR_s | ,132^b^ | ,600 | ,559 | ,164 | ,992 | 1,008 | ,992 |
|  | OH_MDZ_s | ,209^b^ | ,858 | ,407 | ,231 | ,793 | 1,260 | ,793 |
| a. Dependent Variable: DEX | | | | | | | | |
| b. Predictors in the Model: (Constant), OH_TB_s | | | | | | | | |

## DEX [6] with enzymes

| **Model Summary^c,d^** | | | | | |
| --- | --- | --- | --- | --- | --- |
| Model | R | | R Square | Adjusted R Square | Std. Error of the Estimate |
|  | Concentration_ID = Concentration 6 (Selected) | Concentration_ID ~= Concentration 6 (Unselected) |  |  |  |
| 1 | ,743^a^ |  | ,551 | ,519 | 349,51341 |
| 2 | ,808^b^ | ,347 | ,652 | ,599 | 319,41258 |
| a. Predictors: (Constant), CYP3A46_s | | | | | |
| b. Predictors: (Constant), CYP3A46_s, CYP2C33_s | | | | | |
| c. Unless noted otherwise, statistics are based only on cases for which Concentration_ID = Concentration 6. | | | | | |
| d. Dependent Variable: DEX | | | | | |

| **Coefficients^a,b^** | | | | | | | | |
| --- | --- | --- | --- | --- | --- | --- | --- | --- |
| Model | | Unstandardized Coefficients | | Standardized Coefficients | t | Sig. | Collinearity Statistics | |
|  |  | B | Std. Error | Beta |  |  | Tolerance | VIF |
| 1 | (Constant) | 1566,339 | 87,378 |  | 17,926 | ,000 |  |  |
|  | CYP3A46_s | 374,350 | 90,244 | ,743 | 4,148 | ,001 | 1,000 | 1,000 |
| 2 | (Constant) | 1566,339 | 79,853 |  | 19,615 | ,000 |  |  |
|  | CYP3A46_s | 272,379 | 97,800 | ,540 | 2,785 | ,015 | ,711 | 1,406 |
|  | CYP2C33_s | 189,717 | 97,800 | ,376 | 1,940 | ,074 | ,711 | 1,406 |
| a. Dependent Variable: DEX | | | | | | | | |
| b. Selecting only cases for which Concentration_ID = Concentration 6 | | | | | | | | |

| **Excluded Variables^a^** | | | | | | | | |
| --- | --- | --- | --- | --- | --- | --- | --- | --- |
| Model | | Beta In | t | Sig. | Partial Correlation | Collinearity Statistics | | |
|  |  |  |  |  |  | Tolerance | VIF | Minimum Tolerance |
| 1 | CYP3A_s | -,014^b^ | -,045 | ,965 | -,012 | ,365 | 2,740 | ,365 |
|  | CYP2C49_s | ,123^b^ | ,666 | ,517 | ,182 | ,979 | 1,022 | ,979 |
|  | CYP3A22_s | -,203^b^ | -,913 | ,378 | -,245 | ,656 | 1,523 | ,656 |
|  | CYP1A2_s | -,288^b^ | -,860 | ,405 | -,232 | ,291 | 3,441 | ,291 |
|  | CYP2D25_s | ,200^b^ | ,669 | ,515 | ,182 | ,374 | 2,677 | ,374 |
|  | CYP2E1_s | ,151^b^ | ,668 | ,516 | ,182 | ,650 | 1,540 | ,650 |
|  | CYP2C33_s | ,376^b^ | 1,940 | ,074 | ,474 | ,711 | 1,406 | ,711 |
|  | CYP2A19_s | -,221^b^ | -1,141 | ,274 | -,302 | ,838 | 1,194 | ,838 |
| 2 | CYP3A_s | -,128^c^ | -,448 | ,662 | -,128 | ,349 | 2,863 | ,347 |
|  | CYP2C49_s | -,049^c^ | -,249 | ,808 | -,072 | ,735 | 1,360 | ,534 |
|  | CYP3A22_s | -,219^c^ | -1,090 | ,297 | -,300 | ,655 | 1,526 | ,528 |
|  | CYP1A2_s | -,509^c^ | -1,717 | ,112 | -,444 | ,265 | 3,777 | ,265 |
|  | CYP2D25_s | ,024^c^ | ,080 | ,937 | ,023 | ,329 | 3,038 | ,329 |
|  | CYP2E1_s | -,062^c^ | -,255 | ,803 | -,073 | ,485 | 2,060 | ,485 |
|  | CYP2A19_s | -,117^c^ | -,598 | ,561 | -,170 | ,741 | 1,350 | ,528 |
| a. Dependent Variable: DEX | | | | | | | | |
| b. Predictors in the Model: (Constant), CYP3A46_s | | | | | | | | |
| c. Predictors in the Model: (Constant), CYP3A46_s, CYP2C33_s | | | | | | | | |

## DEX [6] with metabolites

| **Model Summary^b,c^** | | | | | |
| --- | --- | --- | --- | --- | --- |
| Model | R | | R Square | Adjusted R Square | Std. Error of the Estimate |
|  | Concentration_ID = Concentration 6 (Selected) | Concentration_ID ~= Concentration 6 (Unselected) |  |  |  |
| 1 | ,610^a^ | ,212 | ,372 | ,327 | 413,67325 |
| a. Predictors: (Constant), OH_MDZ_s | | | | | |
| b. Unless noted otherwise, statistics are based only on cases for which Concentration_ID = Concentration 6. | | | | | |
| c. Dependent Variable: DEX | | | | | |

| **Coefficients^a,b^** | | | | | | | | |
| --- | --- | --- | --- | --- | --- | --- | --- | --- |
| Model | | Unstandardized Coefficients | | Standardized Coefficients | t | Sig. | Collinearity Statistics | |
|  |  | B | Std. Error | Beta |  |  | Tolerance | VIF |
| 1 | (Constant) | 1566,339 | 103,418 |  | 15,146 | ,000 |  |  |
|  | OH_MDZ_s | 307,305 | 106,810 | ,610 | 2,877 | ,012 | 1,000 | 1,000 |
| a. Dependent Variable: DEX | | | | | | | | |
| b. Selecting only cases for which Concentration_ID = Concentration 6 | | | | | | | | |

| **Excluded Variables^a^** | | | | | | | | |
| --- | --- | --- | --- | --- | --- | --- | --- | --- |
| Model | | Beta In | t | Sig. | Partial Correlation | Collinearity Statistics | | |
|  |  |  |  |  |  | Tolerance | VIF | Minimum Tolerance |
| 1 | OH_TB_s | ,009^b^ | ,034 | ,974 | ,009 | ,628 | 1,592 | ,628 |
|  | OH_CZ_s | ,063^b^ | ,173 | ,865 | ,048 | ,361 | 2,769 | ,361 |
|  | OH_CM_s | -,122^b^ | -,524 | ,609 | -,144 | ,876 | 1,142 | ,876 |
|  | PAR_s | ,137^b^ | ,376 | ,713 | ,104 | ,361 | 2,773 | ,361 |
| a. Dependent Variable: DEX | | | | | | | | |
| b. Predictors in the Model: (Constant), OH_MDZ_s | | | | | | | | |

# Independent sample T-test

## Km and Vmax Descriptives

| **Group Statistics** | | | | | | |
| --- | --- | --- | --- | --- | --- | --- |
| Probe_ID | | Sex | N | Mean | Std. Deviation | Std. Error Mean |
| 4-hydroxy-tolbutamide | Vmax | male | 8 | 258,3837 | 254,90279 | 90,12174 |
|  |  | female | 8 | 281,5700 | 76,23877 | 26,95448 |
|  | log_Km | male | 8 | 3,0641 | ,27141 | ,09596 |
|  |  | female | 8 | 3,3239 | ,34076 | ,12048 |
| 6-hydroxy-chorzoxazone | Vmax | male | 8 | 817,5149 | 291,36488 | 103,01304 |
|  |  | female | 8 | 711,0482 | 229,15628 | 81,01898 |
|  | log_Km | male | 8 | 1,7433 | ,13384 | ,04732 |
|  |  | female | 8 | 1,6709 | ,14809 | ,05236 |
| 7-hydroxy-coumarin | Vmax | male | 8 | 294,1372 | 88,92331 | 31,43914 |
|  |  | female | 8 | 313,5077 | 149,35265 | 52,80413 |
|  | log_Km | male | 8 | ,0973 | ,16567 | ,05857 |
|  |  | female | 8 | ,0921 | ,12725 | ,04499 |
| paracetamol | Vmax | male | 8 | 1499,2563 | 450,52054 | 159,28306 |
|  |  | female | 8 | 1309,4169 | 354,37607 | 125,29086 |
|  | log_Km | male | 8 | 1,2876 | ,35026 | ,12384 |
|  |  | female | 8 | 1,1345 | ,16707 | ,05907 |
| 1-hydroxy-midazolam | Vmax | male | 8 | 2274,3177 | 842,86589 | 297,99809 |
|  |  | female | 8 | 2055,2691 | 641,73267 | 226,88676 |
|  | log_Km | male | 8 | 1,3136 | ,13926 | ,04924 |
|  |  | female | 8 | 1,3339 | ,09340 | ,03302 |
| dextrorphan | Vmax | male | 8 | 1752,9830 | 559,11792 | 197,67804 |
|  |  | female | 8 | 1432,4918 | 351,96084 | 124,43695 |
|  | log_Km | male | 8 | ,7105 | ,18975 | ,06709 |
|  |  | female | 8 | ,7843 | ,29305 | ,10361 |

## Km and Vmax Statistics

| **Independent Samples Test** | | | | | | | | | | | |
| --- | --- | --- | --- | --- | --- | --- | --- | --- | --- | --- | --- |
| Probe_ID | | | Levene's Test for Equality of Variances | | t-test for Equality of Means | | | | | | |
|  |  |  | F | Sig. | t | df | Sig. (2-tailed) | Mean Difference | Std. Error Difference | 95% Confidence Interval of the Difference | |
|  |  |  |  |  |  |  |  |  |  | Lower | Upper |
| 4-hydroxy-tolbutamide | Vmax | Equal variances assumed | 4,608 | ,050 | -,246 | 14 | ,809 | -23,18625 | 94,06632 | -224,93844 | 178,56594 |
|  |  | Equal variances not assumed |  |  | -,246 | 8,242 | ,811 | -23,18625 | 94,06632 | -238,99807 | 192,62557 |
|  | log_Km | Equal variances assumed | 1,974 | ,182 | -1,687 | 14 | ,114 | -,25982 | ,15402 | -,59016 | ,07052 |
|  |  | Equal variances not assumed |  |  | -1,687 | 13,333 | ,115 | -,25982 | ,15402 | -,59172 | ,07208 |
| 6-hydroxy-chorzoxazone | Vmax | Equal variances assumed | ,537 | ,476 | ,812 | 14 | ,430 | 106,46667 | 131,05633 | -174,62120 | 387,55454 |
|  |  | Equal variances not assumed |  |  | ,812 | 13,263 | ,431 | 106,46667 | 131,05633 | -176,09277 | 389,02612 |
|  | log_Km | Equal variances assumed | ,454 | ,511 | 1,026 | 14 | ,322 | ,07242 | ,07057 | -,07894 | ,22378 |
|  |  | Equal variances not assumed |  |  | 1,026 | 13,859 | ,322 | ,07242 | ,07057 | -,07909 | ,22392 |
| 7-hydroxy-coumarin | Vmax | Equal variances assumed | 1,619 | ,224 | -,315 | 14 | ,757 | -19,37052 | 61,45483 | -151,17801 | 112,43698 |
|  |  | Equal variances not assumed |  |  | -,315 | 11,409 | ,758 | -19,37052 | 61,45483 | -154,04264 | 115,30161 |
|  | log_Km | Equal variances assumed | ,540 | ,475 | ,070 | 14 | ,945 | ,00519 | ,07386 | -,15322 | ,16360 |
|  |  | Equal variances not assumed |  |  | ,070 | 13,127 | ,945 | ,00519 | ,07386 | -,15421 | ,16459 |
| paracetamol | Vmax | Equal variances assumed | ,886 | ,362 | ,937 | 14 | ,365 | 189,83938 | 202,65462 | -244,81155 | 624,49030 |
|  |  | Equal variances not assumed |  |  | ,937 | 13,264 | ,366 | 189,83938 | 202,65462 | -247,08474 | 626,76349 |
|  | log_Km | Equal variances assumed | 10,602 | ,006 | 1,116 | 14 | ,283 | ,15314 | ,13720 | -,14113 | ,44741 |
|  |  | Equal variances not assumed |  |  | 1,116 | 10,028 | ,290 | ,15314 | ,13720 | -,15245 | ,45872 |
| 1-hydroxy-midazolam | Vmax | Equal variances assumed | ,171 | ,686 | ,585 | 14 | ,568 | 219,04864 | 374,54034 | -584,26049 | 1022,35777 |
|  |  | Equal variances not assumed |  |  | ,585 | 13,074 | ,569 | 219,04864 | 374,54034 | -589,62888 | 1027,72616 |
|  | log_Km | Equal variances assumed | ,497 | ,492 | -,341 | 14 | ,738 | -,02023 | ,05929 | -,14739 | ,10692 |
|  |  | Equal variances not assumed |  |  | -,341 | 12,237 | ,739 | -,02023 | ,05929 | -,14913 | ,10866 |
| dextrorphan | Vmax | Equal variances assumed | 2,894 | ,111 | 1,372 | 14 | ,192 | 320,49113 | 233,58330 | -180,49524 | 821,47749 |
|  |  | Equal variances not assumed |  |  | 1,372 | 11,795 | ,196 | 320,49113 | 233,58330 | -189,42706 | 830,40932 |
|  | log_Km | Equal variances assumed | ,198 | ,663 | -,598 | 14 | ,559 | -,07385 | ,12343 | -,33858 | ,19088 |
|  |  | Equal variances not assumed |  |  | -,598 | 11,992 | ,561 | -,07385 | ,12343 | -,34280 | ,19510 |

## Abundance descriptives

| **Group Statistics** | | | | | |
| --- | --- | --- | --- | --- | --- |
|  | Sex | N | Mean | Std. Deviation | Std. Error Mean |
| log_CYP3A | 1,00 | 8 | ,7167 | ,12062 | ,04265 |
|  | 2,00 | 8 | ,6571 | ,10471 | ,03702 |
| log_CYP2C49 | 1,00 | 8 | ,7994 | ,28981 | ,10246 |
|  | 2,00 | 8 | ,9551 | ,41166 | ,14554 |
| log_CYP3A22 | 1,00 | 8 | ,9818 | ,16984 | ,06005 |
|  | 2,00 | 8 | ,8980 | ,19226 | ,06797 |
| log_CYP1A2 | 1,00 | 8 | ,7588 | ,12229 | ,04324 |
|  | 2,00 | 8 | ,7048 | ,11267 | ,03984 |
| log_CYP2D25 | 1,00 | 8 | 1,6140 | ,17006 | ,06013 |
|  | 2,00 | 8 | 1,5599 | ,13845 | ,04895 |
| log_CYP3A46 | 1,00 | 8 | ,6838 | ,22959 | ,08117 |
|  | 2,00 | 8 | ,6221 | ,16320 | ,05770 |
| log_CYP2E1 | 1,00 | 8 | 1,2827 | ,14960 | ,05289 |
|  | 2,00 | 8 | 1,2507 | ,12840 | ,04540 |
| log_CYP2C33 | 1,00 | 8 | ,6615 | ,19648 | ,06947 |
|  | 2,00 | 8 | ,6533 | ,18826 | ,06656 |
| log_CYP2A19 | 1,00 | 8 | 1,6338 | ,16723 | ,05913 |
|  | 2,00 | 8 | 1,6114 | ,22979 | ,08124 |

## Abundance Statistics

| **Independent Samples Test** | | | | | | | | | | |
| --- | --- | --- | --- | --- | --- | --- | --- | --- | --- | --- |
|  | | Levene's Test for Equality of Variances | | t-test for Equality of Means | | | | | | |
|  |  | F | Sig. | t | df | Sig. (2-tailed) | Mean Difference | Std. Error Difference | 95% Confidence Interval of the Difference | |
|  |  |  |  |  |  |  |  |  | Lower | Upper |
| log_CYP3A | Equal variances assumed | ,160 | ,695 | 1,056 | 14 | ,309 | ,05962 | ,05647 | -,06150 | ,18075 |
|  | Equal variances not assumed |  |  | 1,056 | 13,729 | ,309 | ,05962 | ,05647 | -,06172 | ,18097 |
| log_CYP2C49 | Equal variances assumed | 1,535 | ,236 | -,875 | 14 | ,396 | -,15575 | ,17799 | -,53751 | ,22601 |
|  | Equal variances not assumed |  |  | -,875 | 12,570 | ,398 | -,15575 | ,17799 | -,54163 | ,23012 |
| log_CYP3A22 | Equal variances assumed | ,360 | ,558 | ,924 | 14 | ,371 | ,08380 | ,09070 | -,11072 | ,27833 |
|  | Equal variances not assumed |  |  | ,924 | 13,790 | ,371 | ,08380 | ,09070 | -,11100 | ,27860 |
| log_CYP1A2 | Equal variances assumed | ,018 | ,894 | ,918 | 14 | ,374 | ,05397 | ,05879 | -,07212 | ,18007 |
|  | Equal variances not assumed |  |  | ,918 | 13,907 | ,374 | ,05397 | ,05879 | -,07220 | ,18014 |
| log_CYP2D25 | Equal variances assumed | 1,128 | ,306 | ,697 | 14 | ,497 | ,05407 | ,07753 | -,11223 | ,22036 |
|  | Equal variances not assumed |  |  | ,697 | 13,447 | ,497 | ,05407 | ,07753 | -,11287 | ,22100 |
| log_CYP3A46 | Equal variances assumed | ,761 | ,398 | ,619 | 14 | ,546 | ,06162 | ,09959 | -,15198 | ,27521 |
|  | Equal variances not assumed |  |  | ,619 | 12,635 | ,547 | ,06162 | ,09959 | -,15416 | ,27740 |
| log_CYP2E1 | Equal variances assumed | ,320 | ,581 | ,460 | 14 | ,653 | ,03203 | ,06970 | -,11746 | ,18153 |
|  | Equal variances not assumed |  |  | ,460 | 13,685 | ,653 | ,03203 | ,06970 | -,11779 | ,18185 |
| log_CYP2C33 | Equal variances assumed | ,018 | ,895 | ,085 | 14 | ,933 | ,00819 | ,09621 | -,19815 | ,21453 |
|  | Equal variances not assumed |  |  | ,085 | 13,974 | ,933 | ,00819 | ,09621 | -,19819 | ,21457 |
| log_CYP2A19 | Equal variances assumed | 1,415 | ,254 | ,223 | 14 | ,827 | ,02239 | ,10048 | -,19311 | ,23790 |
|  | Equal variances not assumed |  |  | ,223 | 12,791 | ,827 | ,02239 | ,10048 | -,19504 | ,23983 |
